# Trends in lung cancer incidence by gender, histological type and stage at diagnosis in Japan, 1993 to 2015: A multiple imputation approach

**DOI:** 10.1002/ijc.33962

**Published:** 2022-02-22

**Authors:** Phuong The Nguyen, Kota Katanoda, Eiko Saito, Megumi Hori, Tomio Nakayama, Tomohiro Matsuda

**Affiliations:** ^1^ Graduate School of Public Health St. Luke's International University Tokyo Japan; ^2^ National Cancer Center Institute for Cancer Control Tokyo Japan; ^3^ Institute for Global Health Policy Research National Center for Global Health and Medicine Tokyo Japan; ^4^ School of Nursing University of Shizuoka Shizuoka Japan

**Keywords:** adenocarcinoma, histological type, squamous cell carcinoma, stage at diagnosis

## Abstract

Continued decrease in smoking prevalence and increasing use of sensitive diagnostic procedures necessitate updated monitoring of trends in lung cancer incidence in Japan. We analyzed histology‐ and stage‐specific trends in 1993 to 2015 using data from 62 870 diagnosed cases from the Monitoring of Cancer Incidence in Japan project. After applying a multiple imputation approach to impute missing/unknown values of stage and histology, we estimated age‐standardized incidence rates and applied joinpoint regression analyses. We observed long‐term growth trends in adenocarcinoma (ADC) and localized cancer among both genders, long‐term declining trends among men and leveling‐off trends among women in small‐cell carcinoma (SMC) and squamous cell carcinoma (SQC). Stratifying by gender, we observed an increase in localized ADC with average annual percentage changes (AAPC) of 4.5 (95% confidence interval: 3.9 to 5.0) and 5.7 (5.0 to 6.4), a decrease in regional ADC with AAPC of −1.5 (−2.5 to −0.6) and −2.3 (−4.6 to 0.0), but an increase in distant ADC with AAPC of 1.5 (1.1 to 1.9) and 1.6 (0.9 to 2.3) among males and females, respectively. Additionally, increasing trends in female‐to‐male incidence rate ratios were observed in localized ADC with significantly above one in the most recent diagnosis period. Our results revealed evidence for a partial shift from advanced to early cancer stage, which may suggest the modest effectiveness of nationwide organized screening programs. The observed increasing localized and distant ADC may be linked to improved diagnostic procedures, especially for metastasis detection. Further investigation is needed for more accurate quantification of these factors.

## Graphical abstract

What's new?

In Japan, the combination of reduced smoking prevalence and widespread clinical use of sensitive diagnostic tests has warranted re‐evaluation of trends in lung cancer incidence. Here, comprehensive analyses of lung cancer incidence in Japan were performed using multiple imputation techniques to reduce bias from missing or unknown data. It revealed long‐term increasing trends in adenocarcinoma among men and women, while small cell carcinoma and squamous cell carcinoma showed decreasing trends among men, with leveling off among women. Stratified analyses showed long‐term increasing trends in localized and distant adenocarcinoma among both genders and in distant small cell carcinoma among women. The findings suggest that recent increases in the use of improved diagnostic strategies are impacting trends in lung cancer incidence in Japan.

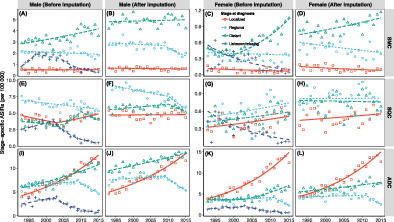

**Abbreviations**
AAPC: average annual percentage change
ADC: adenocarcinoma
APC: annual percentage change
ASIR: age‐standardized incidence rate
DCN: death certificate notification
DCO: death certificate only
FM‐IRR: female‐to‐male incidence rate ratio
LAC: large cell carcinoma
MCIJ: Monitoring of Cancer Incidence in Japan
MI: multiple imputation
MICE: multivariate imputation by chained equations
NCI: National Cancer Institute
NSCLC: nonsmall cell lung cancer
SE: standard error
SEER: Surveillance, Epidemiology and End Results Program
SMC: small cell carcinoma
SQC: squamous cell carcinoma

## 1 INTRODUCTION

Globally, lung cancer is the leading cause of cancer mortality with 1.8 million deaths and the second most common cancer with 2.2 million new cases in 2020.^1^ Asia is the most impacted region by lung cancer, accounting for 60% of the global incidence, mortality rates and 5‐year prevalence in both sexes.^1^ In Japan, lung cancer is among the top three most common cancers (after colon and stomach cancers) with 130 000 new cases and the leading cause of cancer death with 75 600 deaths in 2020.^2^ A recent study of lung cancer incidence and mortality trends in 38 countries demonstrated different patterns between genders globally. While most countries show decreasing or stabilizing incidences and decreasing mortality among men, nearly half of the 38 counties show increasing or stabilizing incidences and increasing or stabilizing mortality among women.^3^ In Japan, previous work has revealed a recent decrease in both lung cancer incidence and mortality rate among men, and an increase in incidence and decrease in mortality among women.^4^

Lung cancers are classified into histological types for determining treatment strategies and projecting long‐term outcomes.^5^ Three main histological types of lung cancer are small cell carcinoma (SMC) and nonsmall cell carcinomas including squamous cell carcinoma (SQC) and adenocarcinoma (ADC).^6^ The most recent study of histology‐specific trends in lung cancer in Japan showed a decreasing trend in SMC and SQC and increasing trends in ADC among both genders.^7^ However, this previous work only examined cancer incidence in a specific prefecture from 1975 to 2008 and did not analyze the combination of the stage‐ and histology‐specific lung cancer trends using data from multiple areas, which is critical for assessing the probability of successful primary and secondary prevention strategies.

Tobacco smoking is the leading preventable cause of the vast majority of lung cancer cases among men around the world (80%)^8^ and in Japan (67.5%).^9^ There are different magnitudes of association and divergent attributable fractions of tobacco smoking to different histological types. For instance, SMC and SQC are more strongly associated with smoking behaviors than ADC, which is predominantly reported among never smokers and young Asian women).^10^, ^11^ Decreasing trends in smoking prevalence in Japan over the 50 years through 2018 among both men and women (from 82.3% and 15.5% in 1965 to 27.8% and 8.7% in 2018, respectively) may have potentially influenced the histological distribution of lung cancer.^12^ Further, smoking behaviors are critical criteria for risk assessment in lung cancer screening guidelines, which target asymptomatic patients and recommend providing chest X‐ray examinations for non‐high‐risk groups and combining chest X‐ray and sputum cytology for high‐risk groups.^13^ While low‐dose computed tomography (LDCT) is not currently recommended for organized screening, this highly sensitive diagnostic procedure has been widely used in clinical practice. Hence, the introduction of screening and diagnostic procedures may have additionally altered the stage distribution and created a potential risk of overdiagnosis (detection of cases that would never cause clinical symptoms or death).^14^, ^15^

One of the challenges of long‐term trend analysis of population‐based cancer incidence data is the presence of nonspecific codes for histological types and missing/unknown data on stage at diagnosis from earlier diagnosis periods. To address this issue, multiple imputation (MI) techniques have been used to handle missing values and code changes in cancer registry data in multiple countries, including Japan.^16^, ^17^, ^18^ Thus, we aimed to use MI to reduce bias due to missing/unknown data and provide comprehensive and the most up‐to‐date analyses of lung cancer incidence using histology‐ and stage‐specific trends in Japan from 1993 to 2015.

## 2 MATERIALS AND METHODS

### 2.1 Data sources

We obtained cancer incidence data from the Monitoring of Cancer Incidence in Japan (MCIJ) project. We selected three qualified population‐based cancer registries in Japan (Yamagata, Fukui and Nagasaki prefectures) to ensure the quality and generalizability of long‐term trends in incidence rates to the Japanese population.^19^, ^20^ We defined lung cancer based on the International Statistical Classification of Diseases and Related Health Problems 10th Revision (ICD‐10) code C34 Malignant neoplasm of bronchus and lung. Finally, we extracted data from 62 870 lung cancer cases diagnosed from 1993 to 2015.

### 2.2 Histology‐ and stage‐specific classifications

We categorized lung cancer into six histological types: SMC, SQC, ADC, large cell carcinoma (LAC), other nonsmall cell lung cancer (NSCLC) and other specified and unspecified types listed in the International Classification of Diseases for Oncology (ICD‐O) following the National Cancer Institute's Surveillance, Epidemiology and End Results Program (NCI's SEER) Cancer Statistics Review.^21^ MCIJ contains two nonspecific codes of 8010 (carcinoma, not otherwise specified) for unspecified carcinomas and 8046 (NSC carcinoma) for unclassifiable cases beyond the exclusion of SMC. Thus, these nonspecific codes 8010 and 8046 were considered missing values. Similarly, we categorized the cancer stage as “Localized,” “Regional” or “Distant” following the NCI's SEER Summary Staging Manual 2000.^22^ Unknown or unreported stages of cancer were considered missing values.

### 2.3 Imputation method

Similar to previous work, we assumed that missing data on histological type and stage at diagnosis were missing at random as demographic and clinical information can randomly determine their distributions.^16^, ^18^ We selected covariates for the imputation models based on (a) demographic characteristics including prefecture, year of diagnosis, age at diagnosis and gender; (b) clinical information including cancer screening status, primary cancer treatment, observation period and vital status during observation time; and (c) data quality indices including the proportions of death certificate notification (DCN%), death certificate only (DCO%) and morphologically verified diagnosis (MV%). Since socioeconomic status was not available in the MCIJ project, we used the Gini coefficient taken from the national survey on family income and expenditure report to control for socioeconomic inequality at the prefecture‐level,^23^ which is associated with cancer incidence and mortality in Japan.^24^ Primary cancer treatments were surgery, radiotherapy, chemotherapy and laparoscopy. We did not include endoscopic treatment, immunotherapy or endocrine therapy due to the small proportion of Japanese lung cancer patients using these treatments (from our data: 0.1%, 0.5% and 0.6%, respectively). The observation period was defined as the time from the year of diagnosis to censor year (2017 for all three prefectures), year of death or last confirmed year of survival, whichever occurred first.

Unknown/unreported data on stage at diagnosis can be imputed as one of three categories: localized, regional and distant. Thus, statistical adjustments can be directly applied to stage at diagnosis using fully conditional specification with the multivariate imputation by chained equations (MICE) algorithm.^25^ In contrast, for histological type, 8010 and 8046 codes are limited to five of six (SMC, SQC, ADC, LAC and other NSCLC) and four of six histological subtypes (SQC, ADC, LAC and other NSCLC), respectively. We thus had to develop a specific amendment to the MICE algorithm to restrict imputed values by constraining the distribution of imputation model parameters. We repeated the procedure 20 times to ensure stability in standard error (SE) estimation and achieve model convergence.^26^

### 2.4 Statistical analysis

We compared the distributions of missing values among imputed variables and selected covariates based on *P*‐values obtained using Pearson's *χ* ^2^ test and Kruskal‐Wallis rank‐sum test. We calculated the age‐standardized incidence rates (ASIRs) of lung cancer per 100 000 person‐years using the “Standard Japanese Population in 1985,” which has been consistently used for Japanese data.^18^, ^19^, ^20^ The ASIRs were computed separately for 20 imputed datasets and the original dataset stratifying by gender, histological type and stage at diagnosis. The pooled ASIRs from 20 imputed datasets were calculated as the arithmetic mean of 20 imputed ASIRs. Following Rubin's rule, the pooled SE was computed from the pooled variance as a combination of within imputation variance (arithmetic mean of the variances of 20 imputed datasets) and between imputation variance (variance of 20 estimated ASIRs over the imputed datasets).^27^ We calculated the 95% confidence intervals (95% CIs) of ASIRs according to the Fay and Feuer method with Tiwari's modification.^28^

We evaluated the histology‐ and stage‐specific trends in lung cancer incidence using the Joinpoint regression program version 4.9.0.0 released by the NCI.^29^ We performed joinpoint regression analysis by fitting log‐linear models, selected optimum model parameters using a grid search with a grid size of 1 year, and detected significant changes in trends using Monte Carlo permutation tests with 4500 iterations, with the following settings: minimum of three observations from a joinpoint to either end of the data and minimum of three observations between two joinpoints.^30^ We calculated annual percentage changes (APC) as the slopes of the log‐linear models at a specific segment between two joinpoints and average annual percentage changes (AAPC) as the slopes of the log‐linear models in the entire period from 1993 to 2015 to evaluate the short‐term and long‐term trends of lung cancer incidence rates, respectively. We estimated the female‐to‐male incidence rate ratios (FM‐IRR) of stage‐ and histology‐specific lung cancer and their 95% CIs from 1993 to 2015 as a subanalysis of gender differences in lung cancer incidence rate in Japan.^31^

### 2.5 Validation and sensitivity analysis

We applied the Kaplan‐Meier log‐rank test to examine the equality in survival probability between the completed dataset (original data excluding missing values) and imputed dataset, stratified by year at diagnosis, gender, stage at diagnosis and histological type. We considered the imputation approach valid if we observed no significant difference in survival probability between the imputed and completed dataset of participants with the same demographic and clinical characteristics.^17^ Additionally, we conducted joinpoint regression analyses for the completed ASIRs (excluding missing values) and compared them with the imputed results as sensitivity analysis.

## 3 RESULTS

Table 1 shows histology‐ and stage‐specific lung cancer cases by period of diagnosis (ie, 1993‐1999, 2000‐2004, 2005‐2009 and 2010‐2015) in three cancer registries in Japan. In histology‐specific lung cancer cases, ADC was the most common type among both men and women in all periods of diagnosis, with overall proportions of 34.8% and 58.4%, respectively in 1993 to 2015. The nonspecific histological codes (8010 and 8046) covered 11% among study participants (6922 cases) and were distributed similarly between gender with 10.5% among men (4638 cases) and 12.2% among women (2284 cases). These proportions fluctuated slightly over time among men (9.5% to 11.2%) and women (10.6% to 13.5%), with no decreasing trends. In stage‐specific lung cancer cases, distant cancers were the most prevalent type among men at all periods of diagnosis, while the localized type became the most widespread cancer among women in the most recent diagnosis period 2010 to 2015 (38.9%). The unconfirmed stage at diagnosis (unknown and missing values) took 23.6% among study participants (14 850 cases), with 23.4% among men (10 289 cases) and 24.3% among women (4561 cases). There were statistically significant reductions in these proportions over time (*P*‐value <.001) among men and women, dropping from 30.3% and 32.5% in 1993 to 1999 to 13.3% and 15.2% in 2010 to 2015, respectively. Table 1 also presents the improvement of data quality over time with the observed decreasing trends in DCN% and DCO% and the increasing trend in MV%.

**TABLE 1:** Histology‐ and stage‐specific cancer cases in three cancer registries^a^ in Japan, 1993 to 2015, by period of diagnosis

Characteristic	Male						Female					
	Overall, N = 44 113^b^	1993‐1999, N = 10 807^b^	2000‐2004, N = 9215^b^	2005‐2009, N = 10 559^b^	2010‐2015, N = 13 532^b^	*P*‐value^c^	Overall, N = 18 757^b^	1993‐1999, N = 4141^b^	2000‐2004, N = 3834^b^	2005‐2009, N = 4498^b^	2010‐2015, N = 6284^b^	*P*‐value^c^
Histological type						<.001						<.001
Small cell carcinoma	4593 (10.4%)	1206 (11.2%)	988 (10.7%)	1060 (10.0%)	1339 (9.9%)		822 (4.4%)	214 (5.2%)	165 (4.3%)	201 (4.5%)	242 (3.9%)	
Squamous cell carcinoma	10 950 (24.8%)	3001 (27.8%)	2273 (24.7%)	2536 (24.0%)	3140 (23.2%)		1223 (6.5%)	318 (7.7%)	250 (6.5%)	271 (6.0%)	384 (6.1%)	
Adenocarcinoma	15 344 (34.8%)	3403 (31.5%)	3103 (33.7%)	3732 (35.3%)	5106 (37.7%)		10 963 (58.4%)	2243 (54.2%)	2161 (56.4%)	2645 (58.8%)	3914 (62.3%)	
Large cell carcinoma	914 (2.1%)	304 (2.8%)	203 (2.2%)	194 (1.8%)	213 (1.6%)		182 (1.0%)	72 (1.7%)	51 (1.3%)	30 (0.7%)	29 (0.5%)	
Other nonsmall cell carcinoma	603 (1.4%)	107 (1.0%)	71 (0.8%)	156 (1.5%)	269 (2.0%)		188 (1.0%)	35 (0.8%)	32 (0.8%)	38 (0.8%)	83 (1.3%)	
Other specified and unspecified types	7071 (16.0%)	1753 (16.2%)	1551 (16.8%)	1737 (16.5%)	2030 (15.0%)		3095 (16.5%)	735 (17.7%)	658 (17.2%)	735 (16.3%)	967 (15.4%)	
8010	4093 (9.3%)	1006 (9.3%)	883 (9.6%)	949 (9.0%)	1255 (9.3%)		2125 (11.3%)	514 (12.4%)	466 (12.2%)	519 (11.5%)	626 (10.0%)	
8046	545 (1.2%)	27 (0.2%)	143 (1.6%)	195 (1.8%)	180 (1.3%)		159 (0.8%)	10 (0.2%)	51 (1.3%)	59 (1.3%)	39 (0.6%)	
Stage at diagnosis						<.001						<.001
Localized	9267 (21.0%)	1766 (16.3%)	1596 (17.3%)	2227 (21.1%)	3678 (27.2%)		5763 (30.7%)	929 (22.4%)	979 (25.5%)	1413 (31.4%)	2442 (38.9%)	
Regional	11 243 (25.5%)	2904 (26.9%)	2290 (24.9%)	2840 (26.9%)	3209 (23.7%)		3367 (18.0%)	852 (20.6%)	730 (19.0%)	853 (19.0%)	932 (14.8%)	
Distant	13 314 (30.2%)	2864 (26.5%)	2359 (25.6%)	3251 (30.8%)	4840 (35.8%)		5066 (27.0%)	1017 (24.6%)	945 (24.6%)	1149 (25.5%)	1955 (31.1%)	
Unknown	5895 (13.4%)	1773 (16.4%)	1642 (17.8%)	1330 (12.6%)	1150 (8.5%)		2434 (13.0%)	707 (17.1%)	613 (16.0%)	568 (12.6%)	546 (8.7%)	
Missing	4394 (10.0%)	1500 (13.9%)	1328 (14.4%)	911 (8.6%)	655 (4.8%)		2127 (11.3%)	636 (15.4%)	567 (14.8%)	515 (11.4%)	409 (6.5%)	
Death certificate notification	8433 (19.1%)	2564 (23.7%)	2356 (25.6%)	2107 (20.0%)	1406 (10.4%)	<.001	3652 (19.5%)	1033 (24.9%)	945 (24.6%)	961 (21.4%)	713 (11.3%)	<.001
Death certificate only	4373 (9.9%)	1498 (13.9%)	1323 (14.4%)	899 (8.5%)	653 (4.8%)	<.001	2111 (11.3%)	636 (15.4%)	561 (14.6%)	507 (11.3%)	407 (6.5%)	<.001
MV%	33 341 (75.6%)	8149 (75.4%)	6784 (73.6%)	7940 (75.2%)	10 468 (77.4%)	<.001	13 630 (72.7%)	2937 (70.9%)	2702 (70.5%)	3268 (72.7%)	4723 (75.2%)	<.001
Proportion of autopsy	9 (0.0%)	0 (0.0%)	2 (0.0%)	3 (0.0%)	4 (0.0%)		0 (0.0%)	0 (0.0%)	0 (0.0%)	0 (0.0%)	0 (0.0%)	

Abbreviation: MV, morphological verification.

^a^ The three Japanese cancer registries are in Yamagata, Fukui and Nagasaki prefectures.

^b^ Frequency (%).

^c^ Pearson's chi‐squared test.

Table 2 presents the stage‐specific lung cancer cases stratified by histological type and period of diagnosis in three cancer registries in Japan. In both genders, the most common stage‐specific cancer among SMC, SQC and ADC were distant, regional and localized cancer, respectively. ADC‐localized cancer in 2010 to 2015 accounted for the greatest number of cases among males and females (2024 and 2065) compared to all other histology‐stage‐specific cancers and periods of diagnosis. The distributions of lung cancer cases for all selected covariates are presented in Tables S1 to S3. We observed an increase in the median age at diagnosis, a slight fluctuation in the screening rate, significant changes in cancer treatment in both genders and noticeable improvements in data quality over the diagnosis periods (Table S1). Records of nonspecific histological codes were more frequently found among females, elderly patients in Nagasaki prefecture and patients with a shorter observation period and did not receive primary treatment (Table S2). Missing/unknown data on stage at diagnosis was more frequent among females, patients aged 80 and above, those who lived in Nagasaki prefecture and those diagnosed in 1993 to 2004, the earliest period examined (Table S3).

**TABLE 2:** Stage‐specific lung cancer cases in three cancer registries^a^ in Japan, 1993 to 2015, by period of diagnosis and histological type

Characteristic	Male						Female					
	Overall, N = 44 113^b^	1993‐1999, N = 10 807^b^	2000‐2004, N = 9215^b^	2005‐2009, N = 10 559^b^	2010‐2015, N = 13 532^b^	*P*‐value^c^	Overall, N = 18 757^b^	1993‐1999, N = 4141^b^	2000‐2004, N = 3834^b^	2005‐2009, N = 4498^b^	2010‐2015, N = 6284^b^	*P*‐value^c^
Small cell carcinoma	4593 (100.0%)	1206 (100.0%)	988 (100.0%)	1060 (100.0%)	1339 (100.0%)	<.001	822 (100.0%)	214 (100.0%)	165 (100.0%)	201 (100.0%)	242 (100.0%)	<.001
Localized	326 (7.1%)	81 (6.7%)	76 (7.7%)	70 (6.6%)	99 (7.4%)		53 (6.4%)	18 (8.4%)	7 (4.2%)	13 (6.5%)	15 (6.2%)	
Regional	1284 (28.0%)	355 (29.4%)	259 (26.2%)	333 (31.4%)	337 (25.2%)		229 (27.9%)	67 (31.3%)	48 (29.1%)	56 (27.9%)	58 (24.0%)	
Distant	2247 (48.9%)	514 (42.6%)	409 (41.4%)	508 (47.9%)	816 (60.9%)		390 (47.4%)	74 (34.6%)	58 (35.2%)	104 (51.7%)	154 (63.6%)	
Unknown/missing	736 (16.0%)	256 (21.2%)	244 (24.7%)	149 (14.1%)	87 (6.5%)		150 (18.2%)	55 (25.7%)	52 (31.5%)	28 (13.9%)	15 (6.2%)	
Squamous cell carcinoma	10 950 (100.0%)	3001 (100.0%)	2273 (100.0%)	2536 (100.0%)	3140 (100.0%)	<.001	1223 (100.0%)	318 (100.0%)	250 (100.0%)	271 (100.0%)	384 (100.0%)	<.001
Localized	2826 (25.8%)	720 (24.0%)	523 (23.0%)	640 (25.2%)	943 (30.0%)		280 (22.9%)	70 (22.0%)	50 (20.0%)	65 (24.0%)	95 (24.7%)	
Regional	4081 (37.3%)	1136 (37.9%)	819 (36.0%)	985 (38.8%)	1141 (36.3%)		399 (32.6%)	95 (29.9%)	78 (31.2%)	94 (34.7%)	132 (34.4%)	
Distant	2573 (23.5%)	610 (20.3%)	457 (20.1%)	610 (24.1%)	896 (28.5%)		325 (26.6%)	75 (23.6%)	65 (26.0%)	62 (22.9%)	123 (32.0%)	
Unknown/missing	1470 (13.4%)	535 (17.8%)	474 (20.9%)	301 (11.9%)	160 (5.1%)		219 (17.9%)	78 (24.5%)	57 (22.8%)	50 (18.5%)	34 (8.9%)	
Adenocarcinoma	15 344 (100.0%)	3403 (100.0%)	3103 (100.0%)	3732 (100.0%)	5106 (100.0%)	<.001	10 963 (100.0%)	2243 (100.0%)	2161 (100.0%)	2645 (100.0%)	3914 (100.0%)	<.001
Localized	4880 (31.8%)	790 (23.2%)	865 (27.9%)	1201 (32.2%)	2024 (39.6%)		4886 (44.6%)	758 (33.8%)	854 (39.5%)	1209 (45.7%)	2065 (52.8%)	
Regional	3930 (25.6%)	1027 (30.2%)	857 (27.6%)	1001 (26.8%)	1045 (20.5%)		2195 (20.0%)	576 (25.7%)	500 (23.1%)	562 (21.2%)	557 (14.2%)	
Distant	5006 (32.6%)	1091 (32.1%)	879 (28.3%)	1207 (32.3%)	1829 (35.8%)		2918 (26.6%)	605 (27.0%)	526 (24.3%)	650 (24.6%)	1137 (29.0%)	
Unknown/missing	1528 (10.0%)	495 (14.5%)	502 (16.2%)	323 (8.7%)	208 (4.1%)		964 (8.8%)	304 (13.6%)	281 (13.0%)	224 (8.5%)	155 (4.0%)	
Other (LAC, NSMC and unspecified types)	8588 (100.0%)	2164 (100.0%)	1825 (100.0%)	2087 (100.0%)	2512 (100.0%)	<.001	3465 (100.0%)	842 (100.0%)	741 (100.0%)	803 (100.0%)	1079 (100.0%)	<.001
Localized	1027 (12.0%)	148 (6.8%)	107 (5.9%)	254 (12.2%)	518 (20.6%)		463 (13.4%)	73 (8.7%)	61 (8.2%)	100 (12.5%)	229 (21.2%)	
Regional	1462 (17.0%)	321 (14.8%)	269 (14.7%)	395 (18.9%)	477 (19.0%)		390 (11.3%)	95 (11.3%)	72 (9.7%)	100 (12.5%)	123 (11.4%)	
Distant	2232 (26.0%)	473 (21.9%)	399 (21.9%)	580 (27.8%)	780 (31.1%)		901 (26.0%)	176 (20.9%)	192 (25.9%)	202 (25.2%)	331 (30.7%)	
Unknown/missing	3867 (45.0%)	1222 (56.5%)	1050 (57.5%)	858 (41.1%)	737 (29.3%)		1711 (49.4%)	498 (59.1%)	416 (56.1%)	401 (49.9%)	396 (36.7%)	
Unknown/missing	4638 (100.0%)	1033 (100.0%)	1026 (100.0%)	1144 (100.0%)	1435 (100.0%)	<.001	2284 (100.0%)	524 (100.0%)	517 (100.0%)	578 (100.0%)	665 (100.0%)	<.001
Localized	208 (4.5%)	27 (2.6%)	25 (2.4%)	62 (5.4%)	94 (6.6%)		81 (3.5%)	10 (1.9%)	7 (1.4%)	26 (4.5%)	38 (5.7%)	
Regional	486 (10.5%)	65 (6.3%)	86 (8.4%)	126 (11.0%)	209 (14.6%)		154 (6.7%)	19 (3.6%)	32 (6.2%)	41 (7.1%)	62 (9.3%)	
Distant	1256 (27.1%)	176 (17.0%)	215 (21.0%)	346 (30.2%)	519 (36.2%)		532 (23.3%)	87 (16.6%)	104 (20.1%)	131 (22.7%)	210 (31.6%)	
Unknown/missing	2688 (58.0%)	765 (74.1%)	700 (68.2%)	610 (53.3%)	613 (42.7%)		1517 (66.4%)	408 (77.9%)	374 (72.3%)	380 (65.7%)	355 (53.4%)	

^a^ The three Japanese cancer registries are in Yamagata, Fukui and Nagasaki prefectures.

^b^ Frequency (%).

^c^ Pearson's *χ* ^2^ test.

**FIGURE 1:**
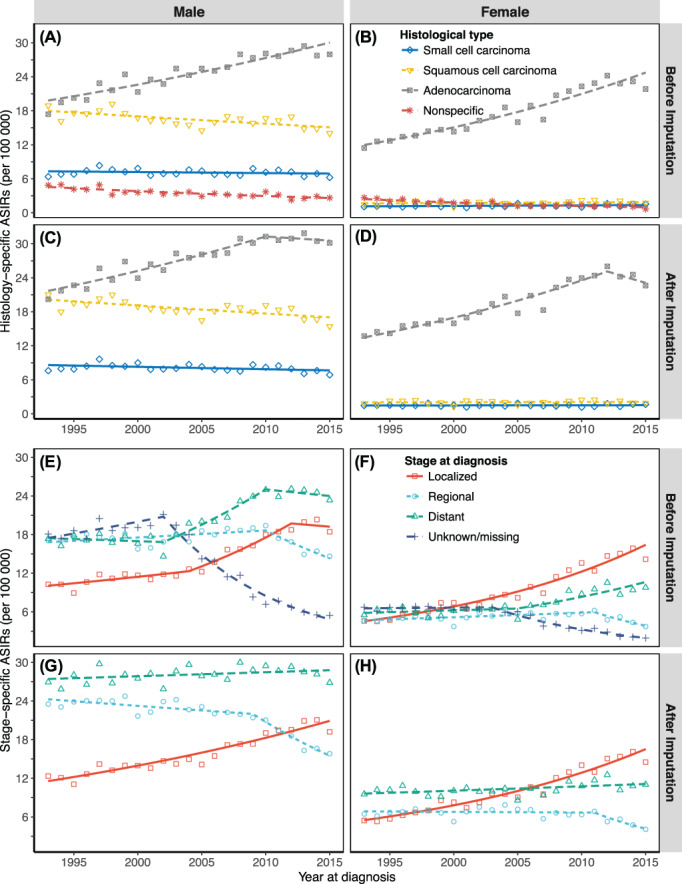
Joinpoint regression analysis of trends in ASIRs in three cancer registries in Japan, 1993 to 2015. (A) Male histology‐specific ASIRs before imputation; (B) Female histology‐specific ASIRs before imputation; (C) Male histology‐specific ASIRs after imputation; (D) Female histology‐specific ASIRs after imputation; (E) Male stage‐specific ASIRs before imputation; (F) Female stage‐specific ASIRs before imputation; (G) Male stage‐specific ASIRs after imputation; (H) Female stage‐specific ASIRs after imputation. ASIRs, age‐standardized incidence rates. The colored dots are the observed rates; lines are the joinpoint model fitted rates. Three Japanese cancer registries are in Yamagata, Fukui and Nagasaki prefectures

Figure 1 shows the histology‐ and stage‐specific trends in ASIRs of lung cancer in Japan with observed data and fitted data from joinpoint regression models. Before imputation, the number of cases with nonspecific histological codes among both males and females started low in 1993 and decreased slightly until 2015. In contrast, the number of cases with unknown/missing stage at diagnosis started highest among all categories in 1993, then decreased to its lowest value in 2015 among both genders. Figures S1 and S2 compare the trends before and after imputing ASIRs of lung cancer by histological type and stage at diagnosis in Japan. While all ASIRs increased after imputation, we observed comparable oscillations in histology‐ and stage‐specific ASIRs of lung cancer before and after imputation. Detailed ASIRs of lung cancer in 1993 to 2015 by gender, histological type and stage at diagnosis are summarized in Tables S4 to S6.

**FIGURE 2:**
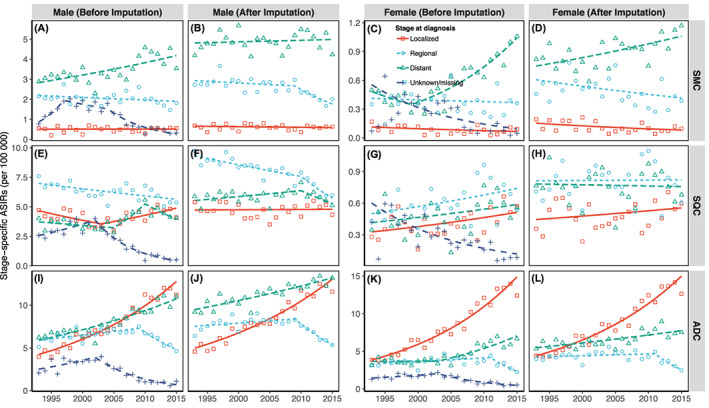
Joinpoint regression analysis of stage‐specific trends in ASIRs of lung cancer by histological type. (A) Male stage‐specific ASIRs of SMC before imputation; (B) Male stage‐specific ASIRs of SMC after imputation; (C) Female stage‐specific ASIRs of SMC before imputation; (D) Female stage‐specific ASIRs of SMC after imputation; (E) Male stage‐specific ASIRs of SQC before imputation; (F) Male stage‐specific ASIRs of SQC after imputation; (G) Female stage‐specific ASIRs of SQC before imputation; (H) Female stage‐specific ASIRs of SQC after imputation; (I) Male stage‐specific ASIRs of ADC before imputation; (J) Male stage‐specific ASIRs of ADC after imputation; (K) Female stage‐specific ASIRs of ADC before imputation; (L) Female stage‐specific ASIRs of ADC after imputation. ADC, adenocarcinoma; ASIRs, age‐standardized incidence rates; SMC, small cell carcinoma; SQC, squamous cell carcinoma. The three Japanese cancer registries are in Yamagata, Fukui and Nagasaki prefectures. The colored dots are the observed rates; colored lines are the joinpoint model‐fitted rates

Table 3 summarizes the results of joinpoint regression analysis of histology‐ and stage‐specific trends in lung cancer ASIRs after imputation. Regarding histology‐specific cancer, there were increasing long‐term trends in ADC among men (AAPC = 1.6, 95% CI: 1.0 to 2.2) and women (AAPC = 2.5, 95% CI: 1.5 to 3.5). While trends in SMC (AAPC = −0.5, 95% CI: −1.0 to −0.1) and SQC (AAPC = −0.8, 95% CI: −1.1 to −0.4) gradually decreased in men, trends in SMC and SQC among women remained unchanged during the observation period. Regarding stage‐specific cancer, we observed long‐term growth trends in localized cancer in men (AAPC = 2.7, 95% CI: 2.4 to 3.1) and women (AAPC = 5.1, 95% CI: 4.6 to 5.7), declining trends in regional cancer in men (AAPC = −2.0, 95% CI: −2.5 to −1.6) and women (AAPC = −2.2, 95% CI: −3.8 to −0.7) and a leveling off in men (AAPC = 0.2, 95% CI: −0.1 to 0.5) and slow accumulation in women (AAPC = 0.7, 95% CI: 0.2 to 1.2) for trends in distant cancer. Localized cancer became the second‐most and most common lung cancer among Japanese men and women, respectively (Figure 1). Although joinpoint regression analysis produced comparable results before and after imputation, we observed changes in short‐term trends in ADC (from increasing trends to leveling‐off trends) among both genders after imputing (Table S7). Table 3 also presents the histology‐stage‐specific trends in imputed ASIRs (complete results are shown in Table S8). Localized ADC showed the most significant growth rate among men (AAPC = 4.5, 95% CI: 3.9 to 5.0) and women (AAPC = 5.7, 95% CI: 5.0 to 6.4). Additionally, following stratification, we observed significant continuous rising trends in distant SMC among women (AAPC = 1.6, 95% CI: 0.7 to 2.5) and in distant ADC among both men (AAPC = 1.5, 95% CI: 1.1 to 1.9) and women (AAPC = 1.6, 95% CI: 0.9 to 2.3). Figure 2 shows the results of joinpoint regression analysis of stage‐specific trends in ASIRs by histological type. We observed significant increasing trends in localized lung ADC among both genders.

**TABLE 3:** Joinpoint regression analysis of trends in imputed ASIRs (after imputation) of lung cancer in three cancer registries in Japan, 1993 to 2015

Category	AAPC (95% CI)	Trend 1		Trend 2	
		Period	APC (95% CI)	Period	APC (95% CI)
*Male*	0.0 (−0.4 to 0.4)	1993‐2011	0.5 (0.3 to 0.7)***	2011‐2015	−2.1 (−4.2 to 0.0)
Histological type					
Small cell carcinoma	−0.5 (−1.0 to −0.1)*	1993‐2015	−0.5 (−1.0 to −0.1)*		
Squamous cell carcinoma	−0.8 (−1.1 to −0.4)***	1993‐2015	−0.8 (−1.1 to −0.4)***		
Adenocarcinoma	1.6 (1.0 to 2.2)***	1993‐2010	2.2 (1.7 to 2.6)***	2010‐2015	−0.5 (−2.8 to 1.9)
Stage at diagnosis					
Localized	2.7 (2.4 to 3.1)***	1993‐2015	2.7 (2.4 to 3.1)***		
Regional	−2.0 (−2.5 to −1.6)***	1993‐2009	−0.6 (−1.0 to −0.2)**	2009‐2015	−5.7 (−7.1 to −4.2)***
Distant	0.2 (−0.1 to 0.5)	1993‐2015	0.2 (−0.1 to 0.5)		
Stratified SMC					
Localized	−0.5 (−1.9 to 0.9)	1993‐2015	−0.5 (−1.9 to 0.9)		
Regional	−2.3 (−3.8 to −0.8)**	1993‐2009	−0.6 (−1.8 to 0.6)	2009‐2015	−6.6 (−11.3 to −1.8)*
Distant	0.2 (−0.5 to 0.8)	1993‐2015	0.2 (−0.5 to 0.8)		
Stratified SQC					
Localized	0.1 (−0.7 to 0.8)	1993‐2015	0.1 (−0.7 to 0.8)		
Regional	−2.2 (−3.1 to −1.3)***	1993‐2010	−1.2 (−1.8 to −0.6)***	2010‐2015	−5.4 (−9.0 to −1.7)**
Distant	−0.2 (−1.3 to 0.9)	1993‐2010	0.8 (0.1 to 1.6)*	2010‐2015	−3.7 (−7.8 to 0.6)
Stratified ADC					
Localized	4.5 (3.9 to 5.0)***	1993‐2015	4.5 (3.9 to 5.0)***		
Regional	−1.5 (−2.5 to −0.6)**	1993‐2009	0.7 (−0.1 to 1.5)	2009‐2015	−7.2 (−10.1 to −4.2)***
Distant	1.5 (1.1 to 1.9)***	1993‐2015	1.5 (1.1 to 1.9)***		
*Female*	1.9 (1.5 to 2.2)***	1993‐2015	1.9 (1.5 to 2.2)***		
Histological type					
Small cell carcinoma	0.2 (−0.7 to 1.0)	1993‐2015	0.2 (−0.7 to 1.0)		
Squamous cell carcinoma	0.2 (−0.8 to 1.2)	1993‐2015	0.2 (−0.8 to 1.2)		
Adenocarcinoma	2.5 (1.5 to 3.5)***	1993‐2012	3.3 (2.8 to 3.9)***	2012‐2015	−2.7 (−9.3 to 4.3)
Stage at diagnosis					
Localized	5.1 (4.6 to 5.7)***	1993‐2015	5.1 (4.6 to 5.7)***		
Regional	−2.2 (−3.8 to −0.7)**	1993‐2011	−0.2 (−1.0 to 0.7)	2011‐2015	−11.1 (−18.2 to −3.3)**
Distant	0.7 (0.2 to 1.2)*	1993‐2015	0.7 (0.2 to 1.2)*		
Stratified SMC					
Localized	−2.8 (−5.2 to −0.4)*	1993‐2015	−2.8 (−5.2 to −0.4)*		
Regional	−1.7 (−3.3 to −0.0)*	1993‐2015	−1.7 (−3.3 to −0.0)*		
Distant	1.6 (0.7 to 2.5)**	1993‐2015	1.6 (0.7 to 2.5)**		
Stratified SQC					
Localized	1.0 (−0.6 to 2.7)	1993‐2015	1.0 (−0.6 to 2.7)		
Regional	0.0 (−1.5 to 1.7)	1993‐2015	0.0 (−1.5 to 1.7)		
Distant	−0.1 (−1.5 to 1.2)	1993‐2015	−0.1 (−1.5 to 1.2)		
Stratified ADC					
Localized	5.7 (5.0 to 6.4)***	1993‐2015	5.7 (5.0 to 6.4)***		
Regional	−2.3 (−4.6 to 0.0)	1993‐2011	0.5 (−0.7 to 1.7)	2011‐2015	−14.1 (−24.5 to −2.2)*
Distant	1.6 (0.9 to 2.3)***	1993‐2015	1.6 (0.9 to 2.3)***		

*Note*: ***, **, * Statistically significant results with *P* < .001, *P* < .01 and *P* < .05, respectively; The three Japanese cancer registries are in Yamagata, Fukui and Nagasaki prefectures.

Abbreviations: AAPC, average annual percentage change; ADC, adenocarcinoma; APC, annual percentage change; ASIRs, age‐standardized incidence rates; CI, confidence interval; SMC, small cell carcinoma; SQC, squamous cell carcinoma.

**FIGURE 3:**
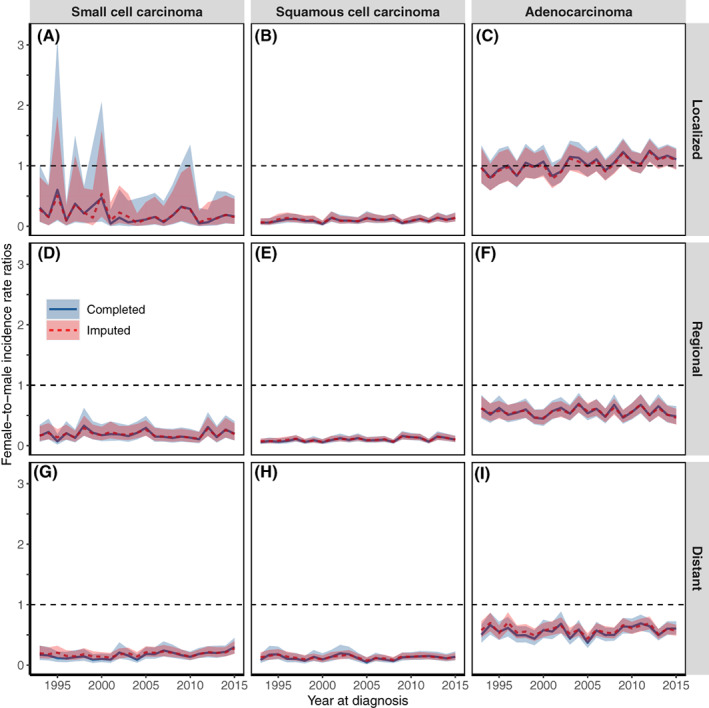
Female‐to‐male IRRs of lung cancer (completed and imputed) in three cancer registries in Japan, 1993 to 2015, by histology and stage combined. (A) Female‐to‐male IRRs of localized SMC; (B) Female‐to‐male IRRs of localized SQC; (C) Female‐to‐male IRRs of localized ADC; (D) Female‐to‐male IRRs of regional SMC; (E) Female‐to‐male IRRs of regional SQC; (F) Female‐to‐male IRRs of regional ADC; (G) Female‐to‐male IRRs of distant SMC; (H) Female‐to‐male IRRs of distant SQC; (I) Female‐to‐male IRRs of distant ADC. ADC, adenocarcinoma; IRRs, incidence rate ratios; SMC, small cell carcinoma; SQC, squamous cell carcinoma. The solid lines are the original trends; the dashed lines are the imputed trends; the shaded areas are the 95% confidence intervals. The three Japanese cancer registries are in Yamagata, Fukui and Nagasaki prefectures

Figure 3 presents the trends in FM‐IRRs for histology‐stage‐specific lung cancer combined in Japan from 1993 to 2015 (separate histology‐ and stage‐specific FM‐IRRs are shown in Figure S3). We observed relatively comparable results before and after imputation, with statistically significantly below‐one FM‐IRRs (women had a lower incidence rate than men) for all subgroups except localized ADC. In contrast, FM‐IRRs for localized ADC reached the threshold of one in most (83%) of the years studied (19 of 23 years) and statistically significant above‐one FM‐IRRs (women had a higher incidence rate than men) were observed in 3 years: 2009, 2012 and 2014. The results of our validation analyses using the Kaplan‐Meier log‐rank test are shown in Tables S9 to S10. We observed no significant difference in survival probability between participants in the imputed and complete datasets with the same demographic and clinical characteristics, proving the validity of our imputation approach.

## 4 DISCUSSION

We applied a multiple imputation approach to provide the most up‐to‐date and comprehensive analysis of lung cancer incidence using histology‐ and stage‐specific trends in Japan from 1993 to 2015. Based on changes in histological distribution, our findings showed that ADC was the most common histology‐specific lung cancer among both genders. Furthermore, by analyzing histology‐stage‐specific trends we observed evidence of a stage shift from regional to localized ADC and a rise in distant ADC among Japanese men and women. Additionally, only localized ADC showed increasing trends in FM‐IRR and above‐one values in the most recent diagnosis period examined. Using high‐quality data from three cancer registries located from northern to southern Japan, our findings have minimal geographic bias and are comparable with national estimates of cancer incidence.^4^, ^19^

In histology‐specific lung cancer, we observed long‐term increasing trends in ADC among both genders that coincided with long‐term decreasing trends in SMC and SQC among men and leveling‐off trends in SMC and SQC among women. These findings are consistent with previous studies on changing histology patterns of lung cancer in Osaka (Japan),^7^ South Korea,^32^ the United States,^33^ the United Kingdom^34^ and other countries.^35^ As incidences of SMC and SQC are highly associated with smoking behavior,^11^ a probable explanation for the reduction in SMC and SQC could be the decline in smoking prevalence, which was most marked in 1965 to 2018 among Japanese men (from 82.3% to 27.8%) than Japanese women (from 15.5% to 8.7%).^12^ Compared to SMC and SQC, the risk factors that primarily contributed to the growing incidence of ADC are unclear. The change from nonfiltered cigarettes to filtered cigarettes by Japanese and Americans from the 1960s may be a potential factor because deeper inhalation can cause peripheral lesions in the lungs, where ADC is more likely to develop than SMC and SQC, which are typically centrally located in the lungs).^36^ Another potential factor that may explain the increasing incidence of ADC is exposure to secondhand smoke.^37^ Sidestream smoke may be more likely to carry tobacco‐specific lung carcinogens to the peripheral regions of the lungs.^38^ Additionally, previous studies have shown that exposure to air pollutants, including fine particles (PM2.5) or nitrogen oxide (NO*x*), might increase the risk of developing lung ADC.^39^, ^40^ However, our findings showed differences in histology‐specific cancer incidence between genders with statistically significant FM‐IRRs, suggesting that general factors like air pollution may not account for all gender‐specific ADC risk in Japan. In addition, we observed that the rate of localized ADC was statistically significantly higher in Japanese women than Japanese men (Figure 3), which is concordant with previous findings in the United States and other countries.^41^, ^42^ A recent study found an association between some reproductive factors (ie, longer fertility span, later age at menopause, natural or surgical menopause) and an increased risk of lung ADC among Japanese women.^43^ While this may explain the rise in lung ADC and the number of unmarried women in Japan in recent years,^44^ further studies using biomarkers for female sex hormones are required to confirm this association.

In stage‐specific cancer, we observed that continuously growing trends in localized cancer incidence coincided with long‐term declining trends in regional cancer incidence among both genders in Japan. This phenomenon suggests a shift toward earlier cancer stages, which have better prognoses and are curable and, according to traditional theory, is an expected outcome of cancer screening.^45^ In fact, there is evidence to suggest that the stage shift may be the result of lung cancer screening programs.^4^, ^46^ In Japan, two lung cancer screening systems are implemented annually including work‐based screening (employer takes responsibility, subjects are employees) and population‐based screening (local municipality government takes responsibility, subjects are the residents not receiving workplace‐based screening).^47^ The screening guideline based on efficacy evaluation initiated in 2007 recommended providing chest X‐ray examinations for non‐high‐risk groups and combining chest X‐ray and sputum cytology for high‐risk groups, which is predominantly classified based on smoking behaviors using the smoking index of 600 or more (calculated as the average number of cigarettes smoked per day multiplied by years of smoking).^13^ The reported increasing lung cancer screening rates among men and women (from 26.7% and 22.9% in 2007 to 47.5% and 37.4% in 2013, respectively) coincide with the observed stage shift and the reducing mortality rates may suggest the modest effectiveness of nationwide organized screening programs in Japan.^4^, ^48^ On the other hand, some researchers have pointed out the predominance of indolent cancers among early detected cases, suggesting the need for stratified analysis that combines histology and stage to better distinguish between actual stage shifts and histology shifts.^49^ After stratification, the only statistically significant increasing trends in localized cancer were in lung ADC among both genders. While lung cancer, the leading cause of cancer death, is aggressive with a very low survival rate (18.1% and 31.2% of 10‐year survival rate among Japanese men and women),^50^ lung ADC is indolent with a good prognosis (>90% 10‐year survival rate).^51^ This increase in localized ADC was followed by a significant decrease in regional cancer, though not by the decrease in distant cancer, still revealed modest evidence for the shift from advanced to early stages of lung ADC. The stage shift phenomenon, if occurred, could be partially linked to the reported increasing lung cancer survival in Japan (from 29.3% in 2005‐2009 to 32.9% in 2010‐2014).^52^ This improvement of lung cancer survival was also reflected in the observed decreasing trends in mortality among both genders.^4^ On the other hand, our stratified analysis showed no stage shift in either gender in the most aggressive lung SMC, which was reported inconsiderable improvements in survival and prognosis among Japanese patients since 1993.^53^

Like many other developed countries, Japan has seen the continued development of imaging devices and technology, such as computed tomography (CT), positron emission tomography (PET) and magnetic resonance imaging (MRI) with increasing sensitivity for diagnosing lung cancer metastases.^54^ Although they are not recommended for population‐based cancer screening as lack scientific evidence,^13^ these highly sensitive diagnostic procedures have been widely used in clinical practice and marginally contributed as opportunistic screening. Specifically, there were 50% to 150% increases in the number of CT scanners and patients using CT services from 2008 to 2017 in hospital and clinic settings in Japan.^55^, ^56^ In the present study, we observed significant increasing trends in localized ADC and distant ADC among both genders, and distant SMC among women. Notably, lung ADC mainly develops in light‐ and never‐smokers,^57^ and is the predominant subtype found by LDCT screening.^58^ Thus, the observed increase in localized and distant ADC could be explained to some extent by the widespread and increasing utilization of those high‐performance imaging devices in Japan.^15^ On the other hand, one of the potential harms from LDCT screening is overdiagnosis (together with false‐positive results and radiation‐induced cancer), which commonly occurs^59^ and has been widely reported in the United Kingdom, Netherlands, Denmark, Germany, Italy and China.^60^ The widespread use of CT in Japan in clinical practice for differential diagnosis of early‐stage cancer could theoretically produce overdiagnosis. However, since the main modality for lung cancer screening in Japan is X‐ray, which reportedly induces less overdiagnosis (~5%),^61^ the observed increase in localized ADC may not be simply attributed to LDCT screening and should be interpreted in a different context. Findings from our study warrant further investigation using empirical or modeling approaches to accurately quantify the overdiagnosis and changes in stage distribution of lung cancer in Japan.^62^

Our joinpoint regression analysis showed several joinpoints occurred in a similar period 2009 to 2012 and mostly focused on regional cancer and ADC. It raises the question of whether this phenomenon has an association with any improvement in screening/diagnostic or change in registration practices in Japan. As PET/CT has been available and in common use in Japan since 2004 to 2005, improvement in screening/diagnostic may be irrelevant to this. Tumor‐node‐metastasis (TNM) classification is used by Japanese cancer registries and was revised in 2009 by the Japanese Society of Lung Cancer, thus could hypothetically contribute to this phenomenon. However, this hypothesis is unsupported by previous findings as trends in overall lung cancer also have joinpoint occurring in 2010.^4^ Nevertheless, providing an evidence‐based explanation is beyond the scope of this article thus requires further investigations.

Some inherent limitations of the present study should be mentioned. First, we selected data from three high‐quality cancer registries to ensure data completeness and timeliness. However, they might differ in lung cancer incidence rate to other registries in Japan, although the validity of the data for trend analyses has been confirmed.^19^, ^20^ Second, as regularly observed in cancer registry data worldwide, data quality indicators such as completeness may improve over time, posing another potential bias in our study. To minimize the influence of such biases on our estimation of trends in lung cancer incidence rates, we included variables of data quality (DCN%, DCO% and MV%) in the imputation models. After imputing the nonspecific histological codes and missing/unknown stage at diagnosis, we observed some changes in ASIRs and trends compared to those before imputation, which were relatively different across stage and histology groups. Specifically, the most influenced histology‐specific trends were ADC, while the most altered stage‐specific trends were found in distant cancer (Figure 1). These findings are understandable as nonspecific histological codes were more often found among females (Table S2), who are more presumably to develop lung ADC.^41^, ^42^ On the other hand, the missing/unknown values of stage at diagnosis were more frequently distributed among older patients (aged 80 and above) and those diagnosed in the earlier period (1993‐2004), who are more probably to be in advanced stages (Table S3). Additionally, our validation analysis using the Kaplan‐Meier log‐rank test confirmed the validity of the multiple imputation approach, with no difference observed in the probability of survival between patients in the imputed and original datasets with the same clinical characteristics.

In summary, our study provides the first and most up‐to‐date estimation of histology‐ and stage‐specific trends in lung cancer incidence in Japan. We showed conspicuous decreasing trends in lung SMC and SQC among men and leveling‐off trends in lung SMC and SQC among women, which may be due to the declining smoking prevalence in Japan. The reported increasing lung cancer screening rates among men and women coincide with the observed stage shift in our study may suggest the modest effectiveness of nationwide organized screening programs in Japan. Furthermore, our stratified analyses showed significant increasing trends in localized and distant ADC among both genders and in distant SMC among women, which may be linked to the widespread and increasing utilization of improved diagnostic procedures (especially for metastasis detection) in the most recent diagnosis period examined. These findings warrant further investigation using empirical or modeling approaches to accurately quantify the multimodal effects of primary/secondary prevention and diagnostic/therapeutic factors for lung cancer in Japan.

## CONFLICT OF INTEREST

KK received JMWH Bayer Grant (1 million JPY) from 1 September 2017 to 31 August 2019, via the Japan Society for Menopause and Women's Health. Other authors have no conflict of interest to declare.

## AUTHOR CONTRIBUTIONS

Study conceptualization: Kota Katanoda, Eiko Saito, Tomohiro Matsuda. Methodology: Kota Katanoda, Eiko Saito, Phuong The Nguyen. Data collection: Kota Katanoda, Eiko Saito, Tomohiro Matsuda. Data analysis and interpretation: Phuong The Nguyen, Megumi Hori. Writing—Original Draft: Phuong The Nguyen, Kota Katanoda. Writing—Review & Editing: Phuong The Nguyen, Kota Katanoda, Eiko Saito, Megumi Hori, Tomio Nakayama, Tomohiro Matsuda. Visualization: Phuong The Nguyen, Kota Katanoda. Supervision: Kota Katanoda. Project administration: Kota Katanoda, Tomohiro Matsuda. The work reported in the article has been performed by the authors, unless clearly specified in the text.

## ETHICS STATEMENT

Our study attained ethical approval (approval number 2019‐202) from the Institutional Review Board of the National Cancer Center Japan.

## Supporting information


Appendix S1 Supporting Information.

## Data Availability

The data that support the findings of our study are available from the corresponding author upon request.
