# Supplementary material for: Trends in lung cancer incidence by gender, histological type and stage at diagnosis in Japan, 1993 to 2015: A multiple imputation approach
Source: Int J Cancer. 2022 Feb 22;151(1):20–32. doi: 10.1002/ijc.33962 (PMC9303961; doi:10.1002/ijc.33962)
Supplement: Supplementary file 1 — Appendix S1 Supporting Information. [file IJC-151-20-s001.pdf]

Supplementary Material

Title: Trends in lung cancer incidence by gender, stage at diagnosis and histological type in Japan, 1993–2015: A multiple imputation approach

Authors: Phuong The Nguyen, Kota Katanoda, Eiko Saito, Megumi Hori, Tomio Nakayama, Tomohiro Matsuda

Table of contents:

Table S1: Selected covariates of male and female lung cancer cases in three cancer registries in Japan, 1993–2015, by periods of diagnosis 2
Table S2: Selected covariates of male and female lung cancer cases in three cancer registries in Japan, 1993–2015, by histological type 4
Table S3: Selected covariates of male and female lung cancer cases in three cancer registries in Japan, 1993–2015, by stage at diagnosis 7
Table S4: ASIRs of lung cancer in three cancer registries in Japan by genders, 1993–2015........ 9
Table S5: Completed and imputed ASIRs of lung cancer in three cancer registries in Japan, 1993–2015, by histological type 10
Table S6: Completed and imputed ASIRs of lung cancer in three cancer registries in Japan, 1993–2015, by stage at diagnosis 14
Table S7: Joinpoint regression analysis of trends in completed ASIRs (before imputation) of lung cancer in three cancer registries in Japan, 1993–2015, by histological type and stage at diagnosis separately. 16
Table S8: Joinpoint regression analysis of trends in completed ASIRs (before imputation) of lung cancer in three cancer registries in Japan, 1993–2015, by histology-stage-combination........ 17
Table S9: Kaplan-Meier log-rank test for equality in survival probability between the completed and imputed data in three cancer registries in Japan, 1993–2015, by stage at diagnosis 18
Table S10: Kaplan-Meier log-rank test for equality in survival probability between the completed and imputed data in three cancer registries in Japan, 1993–2015, by histological type........ 19
Figure S1: Completed and imputed ASIRs (before and after imputation) of lung cancer in three cancer registries in Japan, 1993–2015, by histological type and stage at diagnosis 20
Figure S2: Histology- and stage-specific ASIRs of lung cancer in three cancer registries in Japan, 1993–2015, separately by completed and imputed data (before and after imputation)........ 21
Figure S3: Completed and imputed female-to-male IRRs (before and after imputation) of lung cancer in three cancer registries in Japan, 1993–2015, by histological type and stage at diagnosis separately 22

Table S1: Selected covariates of male and female lung cancer cases in three cancer registries in Japan, 1993–2015, by periods of diagnosis

	Male						Female					
**Characteristic**	**Overall, N = 44,113[1]**	**1993–1999, N = 10,807[1]**	**2000–2004, N = 9,215[1]**	**2005–2009, N = 10,559[1]**	**2010–2015, N = 13,532[1]**	**p-value[2]**	**Overall, N = 18,757[1]**	**1993–1999, N = 4,141[1]**	**2000–2004, N = 3,834[1]**	**2005–2009, N = 4,498[1]**	**2010–2015, N = 6,284[1]**	**p-value[2]**
**Age at diagnosis**	74 [67, 80]	72 [66, 78]	74 [67, 79]	75 [67, 81]	75 [67, 82]	<0.001	75 [66, 82]	73 [64, 80]	74 [66, 82]	76 [67, 83]	76 [67, 83]	<0.001
**Age categories**						<0.001						<0.001
<50 years	1,225 (2.8%)	406 (3.8%)	300 (3.3%)	269 (2.5%)	250 (1.8%)		731 (3.9%)	252 (6.1%)	157 (4.1%)	139 (3.1%)	183 (2.9%)	
50–59 years	4,016 (9.1%)	966 (8.9%)	869 (9.4%)	1,071 (10.1%)	1,110 (8.2%)		1,943 (10.4%)	474 (11.4%)	413 (10.8%)	502 (11.2%)	554 (8.8%)	
60–69 years	11,281 (25.6%)	3,482 (32.2%)	2,138 (23.2%)	2,212 (20.9%)	3,449 (25.5%)		4,054 (21.6%)	1,006 (24.3%)	822 (21.4%)	821 (18.3%)	1,405 (22.4%)	
70–79 years	17,253 (39.1%)	4,110 (38.0%)	4,054 (44.0%)	4,353 (41.2%)	4,736 (35.0%)		6,224 (33.2%)	1,419 (34.3%)	1,340 (35.0%)	1,472 (32.7%)	1,993 (31.7%)	
80+ years	10,338 (23.4%)	1,843 (17.1%)	1,854 (20.1%)	2,654 (25.1%)	3,987 (29.5%)		5,805 (30.9%)	990 (23.9%)	1,102 (28.7%)	1,564 (34.8%)	2,149 (34.2%)	
**Prefecture**						<0.001						<0.001
Yamagata	15,333 (34.8%)	3,760 (34.8%)	3,261 (35.4%)	3,644 (34.5%)	4,668 (34.5%)		5,976 (31.9%)	1,362 (32.9%)	1,210 (31.6%)	1,400 (31.1%)	2,004 (31.9%)	
Fukui	9,653 (21.9%)	2,226 (20.6%)	1,839 (20.0%)	2,442 (23.1%)	3,146 (23.2%)		4,068 (21.7%)	800 (19.3%)	739 (19.3%)	1,019 (22.7%)	1,510 (24.0%)	
Nagasaki	19,127 (43.4%)	4,821 (44.6%)	4,115 (44.7%)	4,473 (42.4%)	5,718 (42.3%)		8,713 (46.5%)	1,979 (47.8%)	1,885 (49.2%)	2,079 (46.2%)	2,770 (44.1%)	
**Gini coefficient**	0.322 ± 0.030 (0.277, 0.376)	0.291 ± 0.011 (0.277, 0.301)	0.307 ± 0.002 (0.304, 0.309)	0.318 ± 0.012 (0.304, 0.331)	0.362 ± 0.013 (0.340, 0.376)	<0.001	0.324 ± 0.030 (0.277, 0.376)	0.291 ± 0.011 (0.277, 0.301)	0.307 ± 0.002 (0.304, 0.309)	0.319 ± 0.012 (0.304, 0.331)	0.361 ± 0.013 (0.340, 0.376)	<0.001
**Observation period**						<0.001						<0.001
0–4 years	37,356 (84.7%)	9,155 (84.7%)	7,583 (82.3%)	8,493 (80.4%)	12,125 (89.6%)		13,837 (73.8%)	3,096 (74.8%)	2,657 (69.3%)	2,938 (65.3%)	5,146 (81.9%)	
5–9 years	4,844 (11.0%)	676 (6.3%)	886 (9.6%)	1,875 (17.8%)	1,407 (10.4%)		3,398 (18.1%)	301 (7.3%)	553 (14.4%)	1,406 (31.3%)	1,138 (18.1%)	
10–14 years	1,156 (2.6%)	438 (4.1%)	527 (5.7%)	191 (1.8%)	0 (0.0%)		888 (4.7%)	328 (7.9%)	406 (10.6%)	154 (3.4%)	0 (0.0%)	
15+ years	757 (1.7%)	538 (5.0%)	219 (2.4%)	0 (0.0%)	0 (0.0%)		634 (3.4%)	416 (10.0%)	218 (5.7%)	0 (0.0%)	0 (0.0%)	
**Screening detected**						<0.001						<0.001
Yes	3,833 (8.7%)	925 (8.6%)	723 (7.8%)	887 (8.4%)	1,298 (9.6%)		2,045 (10.9%)	425 (10.3%)	411 (10.7%)	486 (10.8%)	723 (11.5%)	
No	37,967 (86.1%)	9,047 (83.7%)	7,744 (84.0%)	9,211 (87.2%)	11,965 (88.4%)		15,755 (84.0%)	3,417 (82.5%)	3,165 (82.6%)	3,789 (84.2%)	5,384 (85.7%)	
Unknown/missing	2,313 (5.2%)	835 (7.7%)	748 (8.1%)	461 (4.4%)	269 (2.0%)		957 (5.1%)	299 (7.2%)	258 (6.7%)	223 (5.0%)	177 (2.8%)	
**Surgery**						<0.001						<0.001
Yes	9,009 (20.4%)	3,157 (29.2%)	2,344 (25.4%)	2,032 (19.2%)	1,476 (10.9%)		4,299 (22.9%)	1,439 (34.8%)	1,225 (32.0%)	1,030 (22.9%)	605 (9.6%)	
No	28,274 (64.1%)	5,406 (50.0%)	4,754 (51.6%)	7,062 (66.9%)	11,052 (81.7%)		11,532 (61.5%)	1,863 (45.0%)	1,788 (46.6%)	2,762 (61.4%)	5,119 (81.5%)	
Unknown/missing	6,830 (15.5%)	2,244 (20.8%)	2,117 (23.0%)	1,465 (13.9%)	1,004 (7.4%)		2,926 (15.6%)	839 (20.3%)	821 (21.4%)	706 (15.7%)	560 (8.9%)	
**Chemotherapy**						<0.001						<0.001
Yes	13,703 (31.1%)	2,894 (26.8%)	2,516 (27.3%)	3,490 (33.1%)	4,803 (35.5%)		4,529 (24.1%)	850 (20.5%)	750 (19.6%)	1,113 (24.7%)	1,816 (28.9%)	

Characteristic	Male						Female					
	Overall, N = 44,113[1]	1993–1999, N = 10,807[1]	2000–2004, N = 9,215[1]	2005–2009, N = 10,559[1]	2010–2015, N = 13,532[1]	p-value[2]	Overall, N = 18,757[1]	1993–1999, N = 4,141[1]	2000–2004, N = 3,834[1]	2005–2009, N = 4,498[1]	2010–2015, N = 6,284[1]	p-value[2]
No	23,683 (53.7%)	5,657 (52.3%)	4,619 (50.1%)	5,666 (53.7%)	7,741 (57.2%)		11,085 (59.1%)	2,333 (56.3%)	2,174 (56.7%)	2,667 (59.3%)	3,911 (62.2%)	
Unknown/missing	6,727 (15.2%)	2,256 (20.9%)	2,080 (22.6%)	1,403 (13.3%)	988 (7.3%)		3,143 (16.8%)	958 (23.1%)	910 (23.7%)	718 (16.0%)	557 (8.9%)	
**Radiotherapy**						<0.001						<0.001
Yes	6,839 (15.5%)	1,721 (15.9%)	1,178 (12.8%)	1,593 (15.1%)	2,347 (17.3%)		1,772 (9.4%)	363 (8.8%)	288 (7.5%)	431 (9.6%)	690 (11.0%)	
No	29,967 (67.9%)	6,573 (60.8%)	5,716 (62.0%)	7,500 (71.0%)	10,178 (75.2%)		13,658 (72.8%)	2,707 (65.4%)	2,575 (67.2%)	3,342 (74.3%)	5,034 (80.1%)	
Unknown/missing	7,307 (16.6%)	2,513 (23.3%)	2,321 (25.2%)	1,466 (13.9%)	1,007 (7.4%)		3,327 (17.7%)	1,071 (25.9%)	971 (25.3%)	725 (16.1%)	560 (8.9%)	
**Laparoscopic**						<0.001						<0.001
Yes	3,930 (8.9%)	18 (0.2%)	259 (2.8%)	914 (8.7%)	2,739 (20.2%)		2,865 (15.3%)	5 (0.1%)	192 (5.0%)	653 (14.5%)	2,015 (32.1%)	
No	27,325 (61.9%)	4,188 (38.8%)	5,229 (56.7%)	8,118 (76.9%)	9,790 (72.3%)		10,638 (56.7%)	1,662 (40.1%)	2,162 (56.4%)	3,106 (69.1%)	3,708 (59.0%)	
Unknown/missing	12,858 (29.1%)	6,601 (61.1%)	3,727 (40.4%)	1,527 (14.5%)	1,003 (7.4%)		5,254 (28.0%)	2,474 (59.7%)	1,480 (38.6%)	739 (16.4%)	561 (8.9%)	

[1]Median [IQR] for Age at diagnosis; Mean ± SD (Range) for Gini coefficient; Frequency (%) for all others (calculated by columns)
[2]Pearson's Chi-squared test

Table S2: Selected covariates of male and female lung cancer cases in three cancer registries in Japan, 1993–2015, by histological type

	Male								Female							
**Characteristic**	**Small cell carcinoma, N = 4,593**[1]	**Squamous cell carcinoma, N = 10,950**[1]	**Adenocarcinoma, N = 15,344**[1]	**Large cell carcinoma, N = 914**[1]	**Other non–small cell carcinoma, N = 603**[1]	**Other specified and unspecified types, N = 7,071**[1]	**Unknown/missing, N = 4,638**[1]	**p-value**[2]	**Small cell carcinoma, N = 822**[1]	**Squamous cell carcinoma, N = 1,223**[1]	**Adenocarcinoma, N = 10,963**[1]	**Large cell carcinoma, N = 182**[1]	**Other non–small cell carcinoma, N = 188**[1]	**Other specified and unspecified types, N = 3,095**[1]	**Unknown/missing, N = 2,284**[1]	**p-value**[2]
**Stage at diagnosis**								<0.001								<0.001
Localized	326 (3.5%)	2,826 (30.5%)	4,880 (52.7%)	210 (2.3%)	158 (1.7%)	659 (7.1%)	208 (2.2%)		53 (0.9%)	280 (4.9%)	4,886 (84.8%)	34 (0.6%)	72 (1.2%)	357 (6.2%)	81 (1.4%)	
Regional	1,284 (11.4%)	4,081 (36.3%)	3,930 (35.0%)	295 (2.6%)	199 (1.8%)	968 (8.6%)	486 (4.3%)		229 (6.8%)	399 (11.9%)	2,195 (65.2%)	41 (1.2%)	50 (1.5%)	299 (8.9%)	154 (4.6%)	
Distant	2,247 (16.9%)	2,573 (19.3%)	5,006 (37.6%)	323 (2.4%)	179 (1.3%)	1,730 (13.0%)	1,256 (9.4%)		390 (7.7%)	325 (6.4%)	2,918 (57.6%)	87 (1.7%)	47 (0.9%)	767 (15.1%)	532 (10.5%)	
Unknown/missing	736 (7.2%)	1,470 (14.3%)	1,528 (14.9%)	86 (0.8%)	67 (0.7%)	3,714 (36.1%)	2,688 (26.1%)		150 (3.3%)	219 (4.8%)	964 (21.1%)	20 (0.4%)	19 (0.4%)	1,672 (36.7%)	1,517 (33.3%)	
**Age at diagnosis**	72 [66, 78]	73 [67, 79]	71 [63, 77]	70 [62, 76]	70 [61, 76]	80 [73, 85]	79 [73, 85]	<0.001	73 [67, 79]	75 [68, 80]	71 [63, 78]	71 [63, 78]	70 [61, 79]	84 [77, 88]	84 [77, 89]	<0.001
**Age categories**								<0.001								<0.001
<50 years	80 (6.5%)	190 (15.5%)	714 (58.3%)	61 (5.0%)	47 (3.8%)	73 (6.0%)	60 (4.9%)		16 (2.2%)	24 (3.3%)	583 (79.8%)	18 (2.5%)	19 (2.6%)	41 (5.6%)	30 (4.1%)	
50–59 years	453 (11.3%)	869 (21.6%)	1,997 (49.7%)	129 (3.2%)	96 (2.4%)	237 (5.9%)	235 (5.9%)		76 (3.9%)	81 (4.2%)	1,585 (81.6%)	20 (1.0%)	28 (1.4%)	89 (4.6%)	64 (3.3%)	
60–69 years	1,438 (12.7%)	3,022 (26.8%)	4,736 (42.0%)	284 (2.5%)	185 (1.6%)	980 (8.7%)	636 (5.6%)		222 (5.5%)	278 (6.9%)	3,033 (74.8%)	50 (1.2%)	47 (1.2%)	245 (6.0%)	179 (4.4%)	
70–79 years	1,956 (11.3%)	4,927 (28.6%)	5,769 (33.4%)	352 (2.0%)	204 (1.2%)	2,431 (14.1%)	1,614 (9.4%)		346 (5.6%)	547 (8.8%)	3,942 (63.3%)	67 (1.1%)	60 (1.0%)	729 (11.7%)	533 (8.6%)	
80+ years	666 (6.4%)	1,942 (18.8%)	2,128 (20.6%)	88 (0.9%)	71 (0.7%)	3,350 (32.4%)	2,093 (20.2%)		162 (2.8%)	293 (5.0%)	1,820 (31.4%)	27 (0.5%)	34 (0.6%)	1,991 (34.3%)	1,478 (25.5%)	
**Prefecture**								<0.001								<0.001
Yamagata	1,640 (10.7%)	4,052 (26.4%)	4,845 (31.6%)	301 (2.0%)	144 (0.9%)	4,139 (27.0%)	212 (1.4%)		266 (4.5%)	358 (6.0%)	3,486 (58.3%)	52 (0.9%)	49 (0.8%)	1,727 (28.9%)	38 (0.6%)	
Fukui	917 (9.5%)	2,369 (24.5%)	3,343 (34.6%)	213 (2.2%)	84 (0.9%)	2,586 (26.8%)	141 (1.5%)		161 (4.0%)	280 (6.9%)	2,298 (56.5%)	44 (1.1%)	25 (0.6%)	1,213 (29.8%)	47 (1.2%)	
Nagasaki	2,036 (10.6%)	4,529 (23.7%)	7,156 (37.4%)	400 (2.1%)	375 (2.0%)	346 (1.8%)	4,285 (22.4%)		395 (4.5%)	585 (6.7%)	5,179 (59.4%)	86 (1.0%)	114 (1.3%)	155 (1.8%)	2,199 (25.2%)	
**Gini coefficient**	0.321 ± 0.030 (0.277, 0.376)	0.320 ± 0.030 (0.277, 0.376)	0.324 ± 0.030 (0.277, 0.376)	0.317 ± 0.029 (0.277, 0.376)	0.334 ± 0.029 (0.277, 0.376)	0.317 ± 0.032 (0.277, 0.376)	0.328 ± 0.025 (0.277, 0.376)	<0.001	0.322 ± 0.029 (0.277, 0.376)	0.322 ± 0.029 (0.277, 0.376)	0.326 ± 0.030 (0.277, 0.376)	0.312 ± 0.027 (0.277, 0.376)	0.333 ± 0.030 (0.277, 0.376)	0.318 ± 0.032 (0.277, 0.376)	0.328 ± 0.025 (0.277, 0.376)	<0.001
**Period**								<0.001								<0.001

	Male								Female							
**Characteristic**	**Small cell carcinoma, N = 4,593[1]**	**Squamous cell carcinoma, N = 10,950[1]**	**Adenocarcinoma, N = 15,344[1]**	**Large cell carcinoma, N = 914[1]**	**Other non–small cell carcinoma, N = 603[1]**	**Other specified and unspecified types, N = 7,071[1]**	**Unknown/missing, N = 4,638[1]**	**p-value[2]**	**Small cell carcinoma, N = 822[1]**	**Squamous cell carcinoma, N = 1,223[1]**	**Adenocarcinoma, N = 10,963[1]**	**Large cell carcinoma, N = 182[1]**	**Other non–small cell carcinoma, N = 188[1]**	**Other specified and unspecified types, N = 3,095[1]**	**Unknown/missing, N = 2,284[1]**	**p-value[2]**
1993–1999	1,206 (11.2%)	3,001 (27.8%)	3,403 (31.5%)	304 (2.8%)	107 (1.0%)	1,753 (16.2%)	1,033 (9.6%)		214 (5.2%)	318 (7.7%)	2,243 (54.2%)	72 (1.7%)	35 (0.8%)	735 (17.7%)	524 (12.7%)	
2000–2004	988 (10.7%)	2,273 (24.7%)	3,103 (33.7%)	203 (2.2%)	71 (0.8%)	1,551 (16.8%)	1,026 (11.1%)		165 (4.3%)	250 (6.5%)	2,161 (56.4%)	51 (1.3%)	32 (0.8%)	658 (17.2%)	517 (13.5%)	
2005–2009	1,060 (10.0%)	2,536 (24.0%)	3,732 (35.3%)	194 (1.8%)	156 (1.5%)	1,737 (16.5%)	1,144 (10.8%)		201 (4.5%)	271 (6.0%)	2,645 (58.8%)	30 (0.7%)	38 (0.8%)	735 (16.3%)	578 (12.9%)	
2010–2015	1,339 (9.9%)	3,140 (23.2%)	5,106 (37.7%)	213 (1.6%)	269 (2.0%)	2,030 (15.0%)	1,435 (10.6%)		242 (3.9%)	384 (6.1%)	3,914 (62.3%)	29 (0.5%)	83 (1.3%)	967 (15.4%)	665 (10.6%)	
**Death certificate notification**	547 (6.5%)	912 (10.8%)	1,004 (11.9%)	61 (0.7%)	12 (0.1%)	4,038 (47.9%)	1,859 (22.0%)	<0.001	94 (2.6%)	113 (3.1%)	574 (15.7%)	14 (0.4%)	1 (0.0%)	1,796 (49.2%)	1,060 (29.0%)	<0.001
**Observation period**								<0.001								<0.001
0–4 years	4,283 (11.5%)	8,864 (23.7%)	11,649 (31.2%)	726 (1.9%)	478 (1.3%)	6,855 (18.4%)	4,501 (12.0%)		747 (5.4%)	1,008 (7.3%)	6,707 (48.5%)	148 (1.1%)	129 (0.9%)	2,902 (21.0%)	2,196 (15.9%)	
5–9 years	223 (4.6%)	1,427 (29.5%)	2,699 (55.7%)	127 (2.6%)	101 (2.1%)	167 (3.4%)	100 (2.1%)		47 (1.4%)	136 (4.0%)	2,946 (86.7%)	23 (0.7%)	38 (1.1%)	137 (4.0%)	71 (2.1%)	
10–14 years	55 (4.8%)	429 (37.1%)	572 (49.5%)	33 (2.9%)	11 (1.0%)	30 (2.6%)	26 (2.2%)		16 (1.8%)	50 (5.6%)	749 (84.3%)	6 (0.7%)	12 (1.4%)	44 (5.0%)	11 (1.2%)	
15+ years	32 (4.2%)	230 (30.4%)	424 (56.0%)	28 (3.7%)	13 (1.7%)	19 (2.5%)	11 (1.5%)		12 (1.9%)	29 (4.6%)	561 (88.5%)	5 (0.8%)	9 (1.4%)	12 (1.9%)	6 (0.9%)	
**Screening detected**								<0.001								<0.001
Yes	324 (8.5%)	1,169 (30.5%)	1,868 (48.7%)	93 (2.4%)	57 (1.5%)	283 (7.4%)	39 (1.0%)		41 (2.0%)	75 (3.7%)	1,752 (85.7%)	16 (0.8%)	29 (1.4%)	123 (6.0%)	9 (0.4%)	
No	4,184 (11.0%)	9,683 (25.5%)	13,381 (35.2%)	812 (2.1%)	545 (1.4%)	4,775 (12.6%)	4,587 (12.1%)		763 (4.8%)	1,137 (7.2%)	9,146 (58.1%)	163 (1.0%)	159 (1.0%)	2,116 (13.4%)	2,271 (14.4%)	
Unknown/missing	85 (3.7%)	98 (4.2%)	95 (4.1%)	9 (0.4%)	1 (0.0%)	2,013 (87.0%)	12 (0.5%)		18 (1.9%)	11 (1.1%)	65 (6.8%)	3 (0.3%)	0 (0.0%)	856 (89.4%)	4 (0.4%)	
**Surgery**								<0.001								<0.001
Yes	365 (4.1%)	3,237 (35.9%)	4,589 (50.9%)	353 (3.9%)	217 (2.4%)	116 (1.3%)	132 (1.5%)		56 (1.3%)	291 (6.8%)	3,730 (86.8%)	56 (1.3%)	68 (1.6%)	55 (1.3%)	43 (1.0%)	
No	3,726 (13.2%)	7,003 (24.8%)	9,917 (35.1%)	481 (1.7%)	368 (1.3%)	4,110 (14.5%)	2,669 (9.4%)		677 (5.9%)	847 (7.3%)	6,774 (58.7%)	109 (0.9%)	118 (1.0%)	1,825 (15.8%)	1,182 (10.2%)	
Unknown/missing	502 (7.3%)	710 (10.4%)	838 (12.3%)	80 (1.2%)	18 (0.3%)	2,845 (41.7%)	1,837 (26.9%)		89 (3.0%)	85 (2.9%)	459 (15.7%)	17 (0.6%)	2 (0.1%)	1,215 (41.5%)	1,059 (36.2%)	
**Chemotherapy**								<0.001								<0.001

	Male								Female							
**Characteristic**	**Small cell carcinoma, N = 4,593[1]**	**Squamous cell carcinoma, N = 10,950[1]**	**Adenocarcinoma, N = 15,344[1]**	**Large cell carcinoma, N = 914[1]**	**Other non–small cell carcinoma, N = 603[1]**	**Other specified and unspecified types, N = 7,071[1]**	**Unknown/missing, N = 4,638[1]**	**p-value[2]**	**Small cell carcinoma, N = 822[1]**	**Squamous cell carcinoma, N = 1,223[1]**	**Adenocarcinoma, N = 10,963[1]**	**Large cell carcinoma, N = 182[1]**	**Other non–small cell carcinoma, N = 188[1]**	**Other specified and unspecified types, N = 3,095[1]**	**Unknown/missing, N = 2,284[1]**	**p-value[2]**
Yes	3,035 (22.1%)	3,573 (26.1%)	5,496 (40.1%)	354 (2.6%)	223 (1.6%)	457 (3.3%)	565 (4.1%)		489 (10.8%)	374 (8.3%)	3,229 (71.3%)	60 (1.3%)	51 (1.1%)	150 (3.3%)	176 (3.9%)	
No	1,285 (5.4%)	6,576 (27.8%)	8,874 (37.5%)	491 (2.1%)	363 (1.5%)	3,855 (16.3%)	2,239 (9.5%)		275 (2.5%)	750 (6.8%)	7,014 (63.3%)	104 (0.9%)	131 (1.2%)	1,762 (15.9%)	1,049 (9.5%)	
Unknown/missing	273 (4.1%)	801 (11.9%)	974 (14.5%)	69 (1.0%)	17 (0.3%)	2,759 (41.0%)	1,834 (27.3%)		58 (1.8%)	99 (3.1%)	720 (22.9%)	18 (0.6%)	6 (0.2%)	1,183 (37.6%)	1,059 (33.7%)	
**Radiotherapy**								<0.001								<0.001
Yes	1,135 (16.6%)	2,408 (35.2%)	2,147 (31.4%)	173 (2.5%)	69 (1.0%)	533 (7.8%)	374 (5.5%)		196 (11.1%)	252 (14.2%)	958 (54.1%)	23 (1.3%)	21 (1.2%)	206 (11.6%)	116 (6.5%)	
No	3,010 (10.0%)	7,644 (25.5%)	11,994 (40.0%)	653 (2.2%)	517 (1.7%)	3,720 (12.4%)	2,429 (8.1%)		543 (4.0%)	860 (6.3%)	9,170 (67.1%)	136 (1.0%)	161 (1.2%)	1,678 (12.3%)	1,110 (8.1%)	
Unknown/missing	448 (6.1%)	898 (12.3%)	1,203 (16.5%)	88 (1.2%)	17 (0.2%)	2,818 (38.6%)	1,835 (25.1%)		83 (2.5%)	111 (3.3%)	835 (25.1%)	23 (0.7%)	6 (0.2%)	1,211 (36.4%)	1,058 (31.8%)	
**Laparoscopic**								<0.001								<0.001
Yes	127 (3.2%)	966 (24.6%)	2,579 (65.6%)	93 (2.4%)	125 (3.2%)	13 (0.3%)	27 (0.7%)		16 (0.6%)	100 (3.5%)	2,680 (93.5%)	15 (0.5%)	41 (1.4%)	5 (0.2%)	8 (0.3%)	
No	3,334 (12.2%)	7,242 (26.5%)	9,809 (35.9%)	558 (2.0%)	446 (1.6%)	3,205 (11.7%)	2,731 (10.0%)		617 (5.8%)	854 (8.0%)	6,353 (59.7%)	111 (1.0%)	131 (1.2%)	1,368 (12.9%)	1,204 (11.3%)	
Unknown/missing	1,132 (8.8%)	2,742 (21.3%)	2,956 (23.0%)	263 (2.0%)	32 (0.2%)	3,853 (30.0%)	1,880 (14.6%)		189 (3.6%)	269 (5.1%)	1,930 (36.7%)	56 (1.1%)	16 (0.3%)	1,722 (32.8%)	1,072 (20.4%)	

[1]Median [IQR] for Age at diagnosis; Mean ± SD (Range) for Gini coefficient; Frequency (%) for all others (calculated by rows)
[2]Pearson's Chi-squared test; Kruskal-Wallis rank sum test

Table S3: Selected covariates of male and female lung cancer cases in three cancer registries in Japan, 1993–2015, by stage at diagnosis

	Male					Female				
**Characteristic**	**Localized, N = 9,267[1]**	**Regional, N = 11,243[1]**	**Distant, N = 13,314[1]**	**Unknown/missing, N = 10,289[1]**	**p-value[2]**	**Localized, N = 5,763[1]**	**Regional, N = 3,367[1]**	**Distant, N = 5,066[1]**	**Unknown/missing, N = 4,561[1]**	**p-value[2]**
**Histological type**					<0.001					<0.001
Small cell carcinoma	326 (7.1%)	1,284 (28.0%)	2,247 (48.9%)	736 (16.0%)		53 (6.4%)	229 (27.9%)	390 (47.4%)	150 (18.2%)	
Squamous cell carcinoma	2,826 (25.8%)	4,081 (37.3%)	2,573 (23.5%)	1,470 (13.4%)		280 (22.9%)	399 (32.6%)	325 (26.6%)	219 (17.9%)	
Adenocarcinoma	4,880 (31.8%)	3,930 (25.6%)	5,006 (32.6%)	1,528 (10.0%)		4,886 (44.6%)	2,195 (20.0%)	2,918 (26.6%)	964 (8.8%)	
Large cell carcinoma	210 (23.0%)	295 (32.3%)	323 (35.3%)	86 (9.4%)		34 (18.7%)	41 (22.5%)	87 (47.8%)	20 (11.0%)	
Other non–small cell carcinoma	158 (26.2%)	199 (33.0%)	179 (29.7%)	67 (11.1%)		72 (38.3%)	50 (26.6%)	47 (25.0%)	19 (10.1%)	
Other specified and unspecified types	659 (9.3%)	968 (13.7%)	1,730 (24.5%)	3,714 (52.5%)		357 (11.5%)	299 (9.7%)	767 (24.8%)	1,672 (54.0%)	
Unknown/missing	208 (4.5%)	486 (10.5%)	1,256 (27.1%)	2,688 (58.0%)		81 (3.5%)	154 (6.7%)	532 (23.3%)	1,517 (66.4%)	
**Age at diagnosis**	72 [66, 78]	72 [65, 78]	73 [65, 79]	78 [71, 84]	<0.001	71 [63, 77]	73 [64, 80]	75 [66, 82]	83 [75, 88]	<0.001
**Age categories**					<0.001					<0.001
<50 years	233 (19.0%)	351 (28.7%)	481 (39.3%)	160 (13.1%)		274 (37.5%)	151 (20.7%)	234 (32.0%)	72 (9.8%)	
50–59 years	933 (23.2%)	1,199 (29.9%)	1,424 (35.5%)	460 (11.5%)		805 (41.4%)	422 (21.7%)	553 (28.5%)	163 (8.4%)	
60–69 years	2,693 (23.9%)	3,162 (28.0%)	3,761 (33.3%)	1,665 (14.8%)		1,721 (42.5%)	824 (20.3%)	1,070 (26.4%)	439 (10.8%)	
70–79 years	3,915 (22.7%)	4,602 (26.7%)	4,896 (28.4%)	3,840 (22.3%)		2,140 (34.4%)	1,226 (19.7%)	1,657 (26.6%)	1,201 (19.3%)	
80+ years	1,493 (14.4%)	1,929 (18.7%)	2,752 (26.6%)	4,164 (40.3%)		823 (14.2%)	744 (12.8%)	1,552 (26.7%)	2,686 (46.3%)	
**Prefecture**					<0.001					<0.001
Yamagata	3,077 (20.1%)	3,901 (25.4%)	4,871 (31.8%)	3,484 (22.7%)		1,821 (30.5%)	1,069 (17.9%)	1,700 (28.4%)	1,386 (23.2%)	
Fukui	2,365 (24.5%)	2,442 (25.3%)	3,004 (31.1%)	1,842 (19.1%)		1,498 (36.8%)	675 (16.6%)	1,071 (26.3%)	824 (20.3%)	
Nagasaki	3,825 (20.0%)	4,900 (25.6%)	5,439 (28.4%)	4,963 (25.9%)		2,444 (28.1%)	1,623 (18.6%)	2,295 (26.3%)	2,351 (27.0%)	
**Gini coefficient**	0.328 ± 0.030 (0.277, 0.376)	0.321 ± 0.030 (0.277, 0.376)	0.326 ± 0.031 (0.277, 0.376)	0.314 ± 0.026 (0.277, 0.376)	<0.001	0.329 ± 0.030 (0.277, 0.376)	0.321 ± 0.029 (0.277, 0.376)	0.327 ± 0.031 (0.277, 0.376)	0.317 ± 0.027 (0.277, 0.376)	<0.001
**Period**					<0.001					<0.001
1993–1999	1,766 (16.3%)	2,904 (26.9%)	2,864 (26.5%)	3,273 (30.3%)		929 (22.4%)	852 (20.6%)	1,017 (24.6%)	1,343 (32.4%)	
2000–2004	1,596 (17.3%)	2,290 (24.9%)	2,359 (25.6%)	2,970 (32.2%)		979 (25.5%)	730 (19.0%)	945 (24.6%)	1,180 (30.8%)	
2005–2009	2,227 (21.1%)	2,840 (26.9%)	3,251 (30.8%)	2,241 (21.2%)		1,413 (31.4%)	853 (19.0%)	1,149 (25.5%)	1,083 (24.1%)	
2010–2015	3,678 (27.2%)	3,209 (23.7%)	4,840 (35.8%)	1,805 (13.3%)		2,442 (38.9%)	932 (14.8%)	1,955 (31.1%)	955 (15.2%)	
**Death certificate notification**	308 (3.7%)	887 (10.5%)	1,469 (17.4%)	5,769 (68.4%)	<0.001	101 (2.8%)	252 (6.9%)	542 (14.8%)	2,757 (75.5%)	<0.001
**Observation period**					<0.001					<0.001
0–4 years	5,125 (13.7%)	9,405 (25.2%)	12,997 (34.8%)	9,829 (26.3%)		2,277 (16.5%)	2,511 (18.1%)	4,838 (35.0%)	4,211 (30.4%)	
5–9 years	2,892 (59.7%)	1,392 (28.7%)	257 (5.3%)	303 (6.3%)		2,316 (68.2%)	665 (19.6%)	194 (5.7%)	223 (6.6%)	
10–14 years	762 (65.9%)	279 (24.1%)	30 (2.6%)	85 (7.4%)		658 (74.1%)	126 (14.2%)	23 (2.6%)	81 (9.1%)	
15+ years	488 (64.5%)	167 (22.1%)	30 (4.0%)	72 (9.5%)		512 (80.8%)	65 (10.3%)	11 (1.7%)	46 (7.3%)	
**Screening detected**					<0.001					<0.001
Yes	1,658 (43.3%)	1,166 (30.4%)	745 (19.4%)	264 (6.9%)		1,257 (61.5%)	428 (20.9%)	240 (11.7%)	120 (5.9%)	
No	7,609 (20.0%)	10,077 (26.5%)	12,569 (33.1%)	7,712 (20.3%)		4,506 (28.6%)	2,939 (18.7%)	4,826 (30.6%)	3,484 (22.1%)	
Unknown/missing	0 (0.0%)	0 (0.0%)	0 (0.0%)	2,313 (100.0%)		0 (0.0%)	0 (0.0%)	0 (0.0%)	957 (100.0%)	
**Surgery**					<0.001					<0.001
Yes	4,323 (48.0%)	3,572 (39.6%)	791 (8.8%)	323 (3.6%)		2,632 (61.2%)	1,223 (28.4%)	302 (7.0%)	142 (3.3%)	

	Male					Female				
**Characteristic**	**Localized, N = 9,267[1]**	**Regional, N = 11,243[1]**	**Distant, N = 13,314[1]**	**Unknown/missing, N = 10,289[1]**	**p-value[2]**	**Localized, N = 5,763[1]**	**Regional, N = 3,367[1]**	**Distant, N = 5,066[1]**	**Unknown/missing, N = 4,561[1]**	**p-value[2]**
No	4,812 (17.0%)	7,191 (25.4%)	11,869 (42.0%)	4,402 (15.6%)		3,085 (26.8%)	2,031 (17.6%)	4,579 (39.7%)	1,837 (15.9%)	
Unknown/missing	132 (1.9%)	480 (7.0%)	654 (9.6%)	5,564 (81.5%)		46 (1.6%)	113 (3.9%)	185 (6.3%)	2,582 (88.2%)	
**Chemotherapy**					<0.001					<0.001
Yes	1,358 (9.9%)	5,127 (37.4%)	6,520 (47.6%)	698 (5.1%)		605 (13.4%)	1,408 (31.1%)	2,310 (51.0%)	206 (4.5%)	
No	7,413 (31.3%)	5,718 (24.1%)	6,462 (27.3%)	4,090 (17.3%)		4,844 (43.7%)	1,824 (16.5%)	2,643 (23.8%)	1,774 (16.0%)	
Unknown/missing	496 (7.4%)	398 (5.9%)	332 (4.9%)	5,501 (81.8%)		314 (10.0%)	135 (4.3%)	113 (3.6%)	2,581 (82.1%)	
**Radiotherapy**					<0.001					<0.001
Yes	985 (14.4%)	2,597 (38.0%)	2,944 (43.0%)	313 (4.6%)		324 (18.3%)	491 (27.7%)	901 (50.8%)	56 (3.2%)	
No	7,722 (25.8%)	8,037 (26.8%)	9,803 (32.7%)	4,405 (14.7%)		5,096 (37.3%)	2,681 (19.6%)	3,988 (29.2%)	1,893 (13.9%)	
Unknown/missing	560 (7.7%)	609 (8.3%)	567 (7.8%)	5,571 (76.2%)		343 (10.3%)	195 (5.9%)	177 (5.3%)	2,612 (78.5%)	
**Laparoscopic**					<0.001					<0.001
Yes	2,887 (73.5%)	918 (23.4%)	99 (2.5%)	26 (0.7%)		2,356 (82.2%)	449 (15.7%)	55 (1.9%)	5 (0.2%)	
No	4,894 (17.9%)	7,928 (29.0%)	10,677 (39.1%)	3,826 (14.0%)		2,593 (24.4%)	2,279 (21.4%)	4,176 (39.3%)	1,590 (14.9%)	
Unknown/missing	1,486 (11.6%)	2,397 (18.6%)	2,538 (19.7%)	6,437 (50.1%)		814 (15.5%)	639 (12.2%)	835 (15.9%)	2,966 (56.5%)	

[1]Median [IQR] for Age at diagnosis; Mean ± SD (Range) for Gini coefficient; Frequency (%) for all others (calculated by rows)
[2]Pearson's Chi-squared test; Kruskal-Wallis rank sum test

Table S4: ASIRs of lung cancer in three cancer registries in Japan by genders, 1993–2015

Year at diagnosis	Age-standardized Incidence Rates (per 100,000 person-years)		
	Overall	Male	Female
1993	41.3 (39.4 to 43.2)	62.8 (59.5 to 66.2)	21.5 (19.6 to 23.4)
1994	40.5 (38.7 to 42.4)	61.0 (57.8 to 64.4)	21.6 (19.8 to 23.6)
1995	42.0 (40.2 to 43.9)	63.0 (59.8 to 66.3)	22.6 (20.7 to 24.6)
1996	42.9 (41.0 to 44.8)	63.2 (60.0 to 66.5)	23.9 (22.0 to 25.9)
1997	45.3 (43.4 to 47.2)	68.0 (64.8 to 71.4)	23.8 (21.9 to 25.8)
1998	43.0 (41.2 to 44.9)	64.0 (60.9 to 67.3)	23.2 (21.3 to 25.1)
1999	44.9 (43.1 to 46.8)	66.8 (63.7 to 70.1)	24.4 (22.4 to 26.4)
2000	42.9 (41.2 to 44.8)	63.1 (60.1 to 66.3)	23.6 (21.7 to 25.6)
2001	44.1 (42.3 to 45.9)	64.1 (61.0 to 67.3)	24.8 (22.9 to 26.8)
2002	44.8 (43.0 to 46.6)	64.5 (61.5 to 67.6)	25.7 (23.8 to 27.7)
2003	46.6 (44.8 to 48.5)	67.1 (64.0 to 70.2)	26.7 (24.8 to 28.7)
2004	47.4 (45.6 to 49.3)	67.3 (64.3 to 70.4)	27.8 (25.9 to 29.9)
2005	44.9 (43.1 to 46.7)	65.1 (62.1 to 68.2)	24.8 (23.0 to 26.8)
2006	47.0 (45.2 to 48.8)	65.6 (62.7 to 68.7)	28.1 (26.2 to 30.2)
2007	45.9 (44.1 to 47.8)	66.7 (63.7 to 69.9)	24.6 (22.7 to 26.5)
2008	49.3 (47.4 to 51.2)	69.3 (66.2 to 72.4)	28.7 (26.7 to 30.8)
2009	49.6 (47.7 to 51.5)	67.6 (64.6 to 70.7)	30.8 (28.7 to 33.0)
2010	50.9 (49.0 to 52.8)	69.5 (66.5 to 72.7)	31.2 (29.1 to 33.4)
2011	50.1 (48.2 to 52.0)	67.5 (64.5 to 70.6)	31.5 (29.4 to 33.8)
2012	50.8 (48.9 to 52.7)	67.5 (64.5 to 70.6)	32.9 (30.7 to 35.1)
2013	49.2 (47.3 to 51.0)	65.7 (62.8 to 68.8)	31.2 (29.1 to 33.4)
2014	49.6 (47.7 to 51.5)	65.8 (62.9 to 68.9)	31.7 (29.6 to 33.9)
2015	46.6 (44.8 to 48.4)	61.9 (59.0 to 64.9)	29.6 (27.5 to 31.8)

Notes: ASIRs=Age-standardized incidence rates expressed per 100,000 person-years using the "Standard Japanese Population in 1985"; Three Japanese cancer registries are in Yamagata, Fukui and Nagasaki prefectures.

Table S5: Completed and imputed ASIRs of lung cancer in three cancer registries in Japan, 1993–2015, by histological type

Histological type	Year at diagnosis	Completed ASIRs (per 100,000 person-years)			Imputed ASIRs (per 100,000 person-years)		
		Overall	Male	Female	Overall	Male	Female
Small cell carcinoma	1993	3.6 (3.1 to 4.2)	6.4 (5.3 to 7.5)	1.1 (0.7 to 1.6)	4.4 (3.4 to 5.6)	7.6 (5.8 to 9.7)	1.5 (0.7 to 2.5)
	1994	3.9 (3.3 to 4.5)	6.8 (5.7 to 8.0)	1.2 (0.8 to 1.6)	4.6 (3.7 to 5.7)	7.9 (6.2 to 10.0)	1.5 (0.9 to 2.2)
	1995	3.9 (3.4 to 4.6)	6.8 (5.7 to 8.0)	1.3 (0.9 to 1.8)	4.6 (3.8 to 5.5)	7.9 (6.3 to 9.6)	1.5 (0.9 to 2.3)
	1996	4.1 (3.6 to 4.8)	7.4 (6.4 to 8.6)	1.0 (0.7 to 1.5)	4.8 (4.1 to 5.6)	8.4 (7.0 to 9.9)	1.4 (0.8 to 2.1)
	1997	4.7 (4.1 to 5.4)	8.3 (7.2 to 9.6)	1.2 (0.8 to 1.7)	5.5 (4.5 to 6.6)	9.6 (7.7 to 11.8)	1.5 (0.8 to 2.4)
	1998	4.6 (4.0 to 5.2)	7.6 (6.5 to 8.8)	1.6 (1.1 to 2.2)	5.1 (4.3 to 6.0)	8.5 (7.0 to 10.3)	1.9 (1.1 to 2.8)
	1999	4.1 (3.6 to 4.7)	7.2 (6.2 to 8.3)	1.1 (0.7 to 1.5)	4.8 (3.9 to 5.7)	8.4 (6.9 to 10.0)	1.3 (0.8 to 2.0)
	2000	4.5 (4.0 to 5.1)	7.8 (6.8 to 9.0)	1.3 (0.9 to 1.7)	5.2 (4.4 to 6.1)	9.0 (7.4 to 10.8)	1.5 (1.0 to 2.1)
	2001	3.7 (3.2 to 4.3)	6.6 (5.6 to 7.7)	0.9 (0.6 to 1.3)	4.5 (3.6 to 5.4)	7.8 (6.3 to 9.6)	1.2 (0.6 to 1.9)
	2002	4.0 (3.5 to 4.6)	6.9 (5.9 to 8.0)	1.2 (0.8 to 1.7)	4.6 (3.9 to 5.4)	7.9 (6.5 to 9.5)	1.5 (0.9 to 2.1)
	2003	4.0 (3.5 to 4.6)	6.9 (5.9 to 7.9)	1.2 (0.8 to 1.6)	4.7 (4.0 to 5.5)	8.0 (6.6 to 9.6)	1.5 (0.9 to 2.3)
	2004	4.3 (3.8 to 4.9)	7.6 (6.6 to 8.7)	1.1 (0.7 to 1.5)	5.0 (4.3 to 5.8)	8.7 (7.2 to 10.3)	1.4 (0.9 to 2.0)
	2005	4.5 (4.0 to 5.1)	7.4 (6.4 to 8.5)	1.6 (1.2 to 2.2)	5.1 (4.4 to 5.9)	8.3 (7.0 to 9.8)	1.9 (1.3 to 2.5)
	2006	4.0 (3.5 to 4.6)	6.8 (5.8 to 7.8)	1.2 (0.9 to 1.7)	4.7 (3.9 to 5.5)	7.8 (6.4 to 9.3)	1.4 (1.0 to 2.1)
	2007	4.1 (3.5 to 4.6)	6.8 (5.8 to 7.8)	1.2 (0.8 to 1.7)	4.6 (4.0 to 5.3)	7.7 (6.4 to 9.2)	1.4 (0.9 to 2.1)
	2008	3.9 (3.4 to 4.5)	6.6 (5.7 to 7.6)	1.1 (0.8 to 1.6)	4.5 (3.7 to 5.4)	7.5 (6.1 to 9.0)	1.4 (0.8 to 2.0)
	2009	4.6 (4.1 to 5.2)	7.8 (6.8 to 8.9)	1.3 (0.9 to 1.7)	5.2 (4.5 to 5.9)	8.7 (7.4 to 10.0)	1.5 (1.0 to 2.3)
	2010	4.2 (3.6 to 4.7)	7.2 (6.2 to 8.2)	1.0 (0.6 to 1.4)	4.8 (4.0 to 5.6)	8.2 (6.8 to 9.7)	1.2 (0.7 to 1.7)
	2011	4.4 (3.9 to 5.0)	7.5 (6.6 to 8.6)	1.1 (0.8 to 1.6)	5.0 (4.2 to 5.8)	8.5 (7.2 to 9.9)	1.3 (0.8 to 1.9)
	2012	4.5 (4.0 to 5.1)	7.2 (6.3 to 8.2)	1.6 (1.2 to 2.2)	5.0 (4.3 to 5.7)	7.9 (6.8 to 9.2)	1.8 (1.2 to 2.5)
	2013	3.8 (3.3 to 4.4)	6.4 (5.5 to 7.4)	1.1 (0.8 to 1.6)	4.3 (3.7 to 5.0)	7.1 (6.0 to 8.4)	1.3 (0.9 to 1.8)
	2014	4.4 (3.8 to 4.9)	6.9 (6.0 to 7.9)	1.5 (1.1 to 2.0)	4.8 (4.1 to 5.5)	7.6 (6.3 to 9.1)	1.7 (1.2 to 2.3)
	2015	4.0 (3.5 to 4.6)	6.2 (5.4 to 7.2)	1.6 (1.1 to 2.1)	4.4 (3.7 to 5.1)	6.9 (5.7 to 8.2)	1.7 (1.1 to 2.3)
Squamous cell carcinoma	1993	9.9 (9.0 to 10.8)	18.9 (17.1 to 20.8)	1.5 (1.0 to 2.0)	11.0 (9.9 to 12.3)	21.0 (18.8 to 23.4)	1.8 (1.1 to 2.7)
	1994	8.5 (7.7 to 9.4)	16.2 (14.5 to 17.9)	1.3 (0.9 to 1.9)	9.5 (8.4 to 10.8)	18.0 (15.8 to 20.3)	1.6 (1.0 to 2.4)
	1995	9.6 (8.7 to 10.5)	17.6 (15.9 to 19.4)	2.1 (1.5 to 2.7)	10.6 (9.5 to 11.8)	19.5 (17.3 to 21.9)	2.3 (1.6 to 3.2)
	1996	9.5 (8.6 to 10.4)	17.5 (15.8 to 19.3)	2.0 (1.4 to 2.6)	10.5 (9.4 to 11.6)	19.3 (17.2 to 21.4)	2.3 (1.6 to 3.1)
	1997	9.9 (9.0 to 10.8)	18.0 (16.4 to 19.8)	2.1 (1.6 to 2.7)	11.1 (10.0 to 12.2)	20.3 (18.1 to 22.6)	2.4 (1.6 to 3.3)
	1998	10.1 (9.3 to 11.0)	19.2 (17.5 to 21.0)	1.6 (1.1 to 2.1)	11.1 (9.9 to 12.3)	21.0 (18.7 to 23.4)	1.8 (1.1 to 2.7)
	1999	9.4 (8.6 to 10.3)	17.6 (16.0 to 19.3)	1.7 (1.3 to 2.3)	10.6 (9.5 to 11.8)	19.8 (17.6 to 22.1)	2.0 (1.4 to 2.7)
	2000	8.8 (8.0 to 9.6)	16.8 (15.2 to 18.5)	1.0 (0.7 to 1.4)	9.9 (8.7 to 11.2)	19.0 (16.7 to 21.4)	1.3 (0.7 to 2.0)
	2001	9.0 (8.2 to 9.9)	16.3 (14.8 to 17.9)	2.0 (1.5 to 2.6)	10.3 (9.2 to 11.4)	18.5 (16.4 to 20.8)	2.3 (1.6 to 3.2)
	2002	9.0 (8.2 to 9.9)	16.3 (14.8 to 17.9)	1.9 (1.4 to 2.5)	10.1 (8.9 to 11.3)	18.3 (16.1 to 20.6)	2.2 (1.5 to 3.0)
	2003	8.7 (8.0 to 9.5)	15.7 (14.3 to 17.2)	1.8 (1.3 to 2.3)	10.0 (9.0 to 11.1)	18.1 (16.0 to 20.3)	2.2 (1.5 to 3.0)
	2004	8.6 (7.8 to 9.4)	15.5 (14.1 to 17.0)	1.7 (1.3 to 2.2)	10.0 (8.8 to 11.3)	18.1 (15.9 to 20.5)	2.0 (1.4 to 2.8)
	2005	7.9 (7.2 to 8.6)	14.5 (13.2 to 16.0)	1.3 (0.9 to 1.8)	9.0 (8.1 to 9.9)	16.5 (14.8 to 18.3)	1.6 (1.0 to 2.3)
	2006	8.8 (8.1 to 9.6)	16.0 (14.6 to 17.6)	1.6 (1.1 to 2.1)	10.0 (8.9 to 11.2)	18.1 (16.2 to 20.3)	1.9 (1.2 to 2.7)
	2007	9.5 (8.7 to 10.3)	17.0 (15.5 to 18.6)	1.8 (1.3 to 2.3)	10.7 (9.5 to 11.9)	19.2 (17.1 to 21.4)	2.0 (1.4 to 2.8)
	2008	9.2 (8.4 to 10.0)	16.6 (15.2 to 18.1)	1.4 (1.0 to 1.9)	10.3 (9.2 to 11.5)	18.7 (16.7 to 20.8)	1.7 (1.1 to 2.3)
	2009	9.0 (8.3 to 9.8)	15.8 (14.4 to 17.3)	2.0 (1.5 to 2.5)	10.2 (9.2 to 11.3)	18.0 (16.2 to 19.9)	2.3 (1.7 to 3.0)
	2010	9.6 (8.8 to 10.4)	16.7 (15.2 to 18.2)	2.2 (1.7 to 2.8)	11.0 (10.0 to 12.0)	19.1 (17.3 to 21.1)	2.5 (1.8 to 3.3)
	2011	9.5 (8.7 to 10.3)	16.3 (14.9 to 17.8)	2.2 (1.7 to 2.8)	10.6 (9.6 to 11.6)	18.4 (16.5 to 20.3)	2.5 (1.9 to 3.2)
	2012	9.5 (8.7 to 10.3)	16.9 (15.5 to 18.5)	1.6 (1.2 to 2.1)	10.5 (9.5 to 11.6)	18.7 (16.8 to 20.7)	1.8 (1.3 to 2.5)
	2013	8.7 (7.9 to 9.5)	14.8 (13.5 to 16.3)	2.1 (1.6 to 2.7)	9.7 (8.8 to 10.6)	16.6 (15.1 to 18.3)	2.3 (1.7 to 3.0)
	2014	8.6 (7.8 to 9.4)	14.8 (13.5 to 16.3)	1.7 (1.3 to 2.3)	9.6 (8.6 to 10.6)	16.6 (14.8 to 18.6)	1.9 (1.4 to 2.6)

Histological type	Year at diagnosis	Completed ASIRs (per 100,000 person-years)			Imputed ASIRs (per 100,000 person-years)		
		Overall	Male	Female	Overall	Male	Female
	2015	8.2 (7.5 to 9.0)	14.0 (12.7 to 15.4)	1.7 (1.3 to 2.3)	9.0 (8.0 to 9.9)	15.4 (13.8 to 17.2)	1.9 (1.4 to 2.5)
Adenocarcinoma	1993	14.3 (13.2 to 15.5)	17.4 (15.7 to 19.3)	11.4 (10.1 to 12.9)	16.9 (15.2 to 18.7)	20.2 (17.5 to 23.1)	13.7 (12.0 to 15.6)
	1994	15.9 (14.7 to 17.1)	19.5 (17.6 to 21.4)	12.6 (11.2 to 14.2)	17.9 (16.4 to 19.5)	21.7 (19.4 to 24.2)	14.5 (12.6 to 16.4)
	1995	16.3 (15.2 to 17.5)	20.3 (18.4 to 22.2)	12.7 (11.3 to 14.2)	18.3 (16.6 to 20.0)	22.7 (20.0 to 25.6)	14.2 (12.5 to 16.0)
	1996	16.6 (15.4 to 17.8)	19.9 (18.1 to 21.8)	13.4 (12.0 to 15.0)	18.7 (17.3 to 20.1)	22.0 (19.8 to 24.4)	15.5 (13.8 to 17.3)
	1997	18.2 (16.9 to 19.4)	22.9 (20.9 to 24.9)	13.6 (12.2 to 15.2)	20.7 (19.1 to 22.3)	25.7 (23.3 to 28.2)	15.8 (13.9 to 17.9)
	1998	18.0 (16.8 to 19.2)	21.6 (19.8 to 23.6)	14.4 (12.9 to 16.0)	19.7 (18.2 to 21.2)	23.6 (21.1 to 26.3)	15.9 (14.1 to 17.8)
	1999	19.4 (18.2 to 20.7)	24.4 (22.5 to 26.5)	14.7 (13.1 to 16.3)	21.5 (19.9 to 23.3)	26.9 (24.1 to 29.8)	16.5 (14.8 to 18.3)
	2000	17.8 (16.6 to 19.0)	21.3 (19.5 to 23.2)	14.3 (12.8 to 15.9)	19.9 (18.4 to 21.5)	23.9 (21.5 to 26.5)	16.0 (14.3 to 17.8)
	2001	19.1 (17.9 to 20.4)	23.5 (21.6 to 25.6)	14.8 (13.3 to 16.4)	21.6 (20.2 to 23.2)	26.4 (23.9 to 29.0)	17.0 (15.2 to 18.9)
	2002	19.5 (18.3 to 20.8)	22.7 (20.9 to 24.7)	16.3 (14.7 to 18.0)	21.6 (20.2 to 23.2)	25.4 (23.0 to 27.8)	17.9 (16.2 to 19.9)
	2003	21.2 (19.9 to 22.5)	25.4 (23.5 to 27.5)	16.9 (15.3 to 18.6)	23.8 (22.3 to 25.5)	28.3 (25.8 to 31.0)	19.4 (17.5 to 21.4)
	2004	21.5 (20.2 to 22.8)	24.3 (22.4 to 26.3)	18.6 (16.9 to 20.4)	24.2 (22.6 to 25.7)	27.5 (25.1 to 30.2)	20.7 (18.8 to 22.6)
	2005	20.8 (19.5 to 22.1)	25.5 (23.5 to 27.5)	16.0 (14.5 to 17.6)	23.0 (21.4 to 24.6)	28.1 (25.7 to 30.7)	17.7 (16.0 to 19.5)
	2006	22.1 (20.8 to 23.4)	25.0 (23.2 to 27.1)	18.9 (17.2 to 20.7)	24.5 (23.0 to 26.2)	28.0 (25.5 to 30.7)	20.8 (19.0 to 22.8)
	2007	21.2 (20.0 to 22.5)	25.7 (23.8 to 27.7)	16.4 (14.9 to 18.1)	23.5 (22.0 to 25.1)	28.4 (25.8 to 31.0)	18.3 (16.5 to 20.2)
	2008	24.5 (23.1 to 25.9)	28.0 (25.9 to 30.1)	20.7 (19.0 to 22.6)	26.7 (25.1 to 28.5)	30.9 (28.3 to 33.6)	22.3 (20.3 to 24.3)
	2009	24.6 (23.3 to 26.0)	27.3 (25.4 to 29.4)	21.5 (19.7 to 23.4)	27.1 (25.5 to 28.8)	30.1 (27.7 to 32.7)	23.7 (21.7 to 25.8)
	2010	25.4 (24.0 to 26.8)	28.1 (26.1 to 30.2)	22.3 (20.5 to 24.2)	27.8 (26.2 to 29.5)	31.3 (28.8 to 33.9)	24.0 (22.0 to 26.0)
	2011	25.5 (24.1 to 27.0)	27.6 (25.7 to 29.7)	22.9 (21.0 to 24.9)	27.8 (26.2 to 29.5)	30.6 (28.2 to 33.2)	24.4 (22.4 to 26.5)
	2012	26.7 (25.2 to 28.1)	28.6 (26.6 to 30.8)	24.2 (22.3 to 26.2)	28.7 (27.1 to 30.5)	30.9 (28.6 to 33.4)	26.0 (23.9 to 28.3)
	2013	26.4 (25.0 to 27.9)	29.4 (27.4 to 31.6)	22.8 (20.9 to 24.8)	28.4 (26.8 to 30.0)	31.9 (29.6 to 34.3)	24.2 (22.3 to 26.2)
	2014	25.8 (24.4 to 27.2)	27.7 (25.7 to 29.8)	23.2 (21.3 to 25.2)	27.9 (26.2 to 29.6)	30.5 (28.0 to 33.1)	24.6 (22.6 to 26.6)
	2015	25.2 (23.8 to 26.6)	28.0 (26.0 to 30.1)	21.8 (20.0 to 23.8)	26.7 (25.1 to 28.4)	30.1 (27.8 to 32.7)	22.6 (20.7 to 24.7)
Large cell carcinoma	1993	1.1 (0.8 to 1.4)	1.8 (1.3 to 2.5)	0.4 (0.2 to 0.7)	1.2 (0.8 to 1.8)	2.1 (1.3 to 3.0)	0.5 (0.2 to 0.9)
	1994	1.1 (0.8 to 1.4)	1.9 (1.4 to 2.6)	0.2 (0.1 to 0.5)	1.2 (0.8 to 1.7)	2.1 (1.4 to 3.1)	0.3 (0.1 to 0.6)
	1995	1.0 (0.8 to 1.4)	1.6 (1.1 to 2.3)	0.5 (0.3 to 0.8)	1.2 (0.7 to 1.7)	1.8 (1.1 to 2.8)	0.5 (0.2 to 1.0)
	1996	1.2 (0.9 to 1.5)	2.0 (1.4 to 2.7)	0.4 (0.2 to 0.7)	1.3 (0.9 to 1.8)	2.2 (1.4 to 3.0)	0.5 (0.1 to 1.0)
	1997	1.3 (1.0 to 1.6)	2.0 (1.4 to 2.6)	0.6 (0.3 to 0.9)	1.4 (1.0 to 1.9)	2.2 (1.5 to 3.2)	0.6 (0.3 to 1.0)
	1998	1.1 (0.8 to 1.5)	1.9 (1.4 to 2.6)	0.4 (0.2 to 0.7)	1.2 (0.9 to 1.7)	2.1 (1.4 to 2.9)	0.4 (0.1 to 0.8)
	1999	1.3 (1.0 to 1.6)	2.1 (1.6 to 2.8)	0.5 (0.3 to 0.9)	1.4 (1.0 to 1.9)	2.3 (1.5 to 3.2)	0.6 (0.3 to 1.0)
	2000	0.8 (0.6 to 1.1)	1.2 (0.8 to 1.7)	0.5 (0.2 to 0.8)	0.9 (0.6 to 1.4)	1.4 (0.8 to 2.1)	0.5 (0.2 to 1.0)
	2001	1.0 (0.8 to 1.4)	1.7 (1.2 to 2.3)	0.4 (0.2 to 0.6)	1.2 (0.8 to 1.7)	2.0 (1.3 to 2.9)	0.4 (0.2 to 0.8)
	2002	1.0 (0.8 to 1.4)	1.7 (1.2 to 2.3)	0.4 (0.2 to 0.7)	1.1 (0.8 to 1.6)	1.8 (1.2 to 2.6)	0.4 (0.2 to 0.8)
	2003	1.1 (0.9 to 1.5)	2.0 (1.5 to 2.6)	0.3 (0.1 to 0.5)	1.3 (0.9 to 1.7)	2.2 (1.5 to 3.0)	0.3 (0.1 to 0.7)
	2004	0.9 (0.6 to 1.1)	1.4 (1.0 to 2.0)	0.3 (0.1 to 0.6)	1.0 (0.6 to 1.5)	1.7 (0.9 to 2.6)	0.3 (0.1 to 0.7)
	2005	0.7 (0.5 to 1.0)	1.3 (0.9 to 1.8)	0.1 (0.0 to 0.3)	0.8 (0.5 to 1.2)	1.5 (0.9 to 2.3)	0.2 (0.0 to 0.4)
	2006	0.7 (0.5 to 1.0)	1.1 (0.8 to 1.6)	0.2 (0.1 to 0.5)	0.8 (0.5 to 1.2)	1.3 (0.8 to 2.1)	0.3 (0.1 to 0.6)
	2007	0.9 (0.7 to 1.2)	1.6 (1.1 to 2.2)	0.2 (0.1 to 0.5)	1.0 (0.7 to 1.5)	1.7 (1.1 to 2.7)	0.3 (0.1 to 0.5)
	2008	0.7 (0.5 to 1.0)	1.2 (0.8 to 1.7)	0.2 (0.1 to 0.4)	0.8 (0.5 to 1.2)	1.4 (0.8 to 2.1)	0.2 (0.1 to 0.5)
	2009	1.0 (0.8 to 1.3)	1.8 (1.3 to 2.4)	0.2 (0.1 to 0.4)	1.1 (0.8 to 1.6)	2.0 (1.3 to 2.9)	0.3 (0.1 to 0.6)
	2010	0.9 (0.7 to 1.2)	1.7 (1.2 to 2.3)	0.1 (0.0 to 0.3)	1.0 (0.7 to 1.5)	1.9 (1.2 to 2.8)	0.2 (0.0 to 0.4)
	2011	0.6 (0.4 to 0.9)	1.1 (0.8 to 1.6)	0.1 (0.0 to 0.3)	0.7 (0.4 to 1.1)	1.3 (0.8 to 2.0)	0.1 (0.0 to 0.3)
	2012	0.6 (0.4 to 0.9)	1.0 (0.7 to 1.4)	0.2 (0.1 to 0.4)	0.7 (0.4 to 1.0)	1.1 (0.6 to 1.7)	0.3 (0.1 to 0.5)
	2013	0.7 (0.5 to 0.9)	1.1 (0.8 to 1.5)	0.1 (0.0 to 0.3)	0.7 (0.5 to 1.1)	1.3 (0.8 to 1.9)	0.2 (0.0 to 0.3)
	2014	0.6 (0.4 to 0.8)	1.1 (0.8 to 1.6)	0.0 (0.0 to 0.1)	0.7 (0.4 to 1.1)	1.3 (0.7 to 2.0)	0.0 (0.0 to 0.1)

Histological type	Year at diagnosis	Completed ASIRs (per 100,000 person-years)			Imputed ASIRs (per 100,000 person-years)		
		Overall	Male	Female	Overall	Male	Female
	2015	0.7 (0.5 to 0.9)	1.0 (0.7 to 1.4)	0.3 (0.2 to 0.6)	0.8 (0.5 to 1.1)	1.1 (0.6 to 1.8)	0.4 (0.2 to 0.6)
Other non–small cell carcinoma	1993	0.2 (0.1 to 0.4)	0.2 (0.1 to 0.5)	0.2 (0.0 to 0.4)	0.2 (0.1 to 0.5)	0.3 (0.1 to 0.7)	0.2 (0.0 to 0.5)
	1994	0.5 (0.3 to 0.7)	0.9 (0.5 to 1.3)	0.2 (0.0 to 0.4)	0.5 (0.3 to 0.9)	0.9 (0.5 to 1.6)	0.2 (0.0 to 0.4)
	1995	0.6 (0.4 to 0.9)	0.9 (0.6 to 1.4)	0.3 (0.1 to 0.6)	0.6 (0.4 to 1.0)	1.0 (0.6 to 1.5)	0.3 (0.1 to 0.7)
	1996	0.5 (0.3 to 0.8)	0.8 (0.4 to 1.2)	0.3 (0.1 to 0.5)	0.5 (0.3 to 0.8)	0.8 (0.4 to 1.3)	0.3 (0.1 to 0.6)
	1997	0.5 (0.3 to 0.7)	0.8 (0.5 to 1.3)	0.2 (0.1 to 0.5)	0.5 (0.3 to 0.9)	0.9 (0.4 to 1.4)	0.2 (0.1 to 0.5)
	1998	0.4 (0.2 to 0.6)	0.5 (0.3 to 0.9)	0.3 (0.1 to 0.5)	0.4 (0.2 to 0.7)	0.6 (0.2 to 1.1)	0.3 (0.1 to 0.6)
	1999	0.4 (0.2 to 0.6)	0.7 (0.4 to 1.1)	0.1 (0.0 to 0.3)	0.4 (0.2 to 0.8)	0.7 (0.4 to 1.2)	0.2 (0.0 to 0.4)
	2000	0.4 (0.3 to 0.6)	0.4 (0.2 to 0.8)	0.4 (0.2 to 0.7)	0.5 (0.2 to 0.9)	0.5 (0.2 to 1.1)	0.4 (0.2 to 0.8)
	2001	0.3 (0.2 to 0.5)	0.4 (0.2 to 0.8)	0.2 (0.1 to 0.5)	0.4 (0.1 to 0.7)	0.5 (0.2 to 1.0)	0.3 (0.1 to 0.7)
	2002	0.3 (0.2 to 0.5)	0.6 (0.3 to 0.9)	0.1 (0.0 to 0.3)	0.4 (0.2 to 0.7)	0.7 (0.3 to 1.2)	0.1 (0.0 to 0.3)
	2003	0.4 (0.3 to 0.7)	0.7 (0.4 to 1.0)	0.2 (0.1 to 0.5)	0.5 (0.2 to 0.8)	0.7 (0.3 to 1.3)	0.3 (0.1 to 0.6)
	2004	0.5 (0.3 to 0.7)	0.7 (0.4 to 1.0)	0.3 (0.1 to 0.5)	0.5 (0.3 to 0.8)	0.8 (0.4 to 1.3)	0.3 (0.1 to 0.6)
	2005	0.8 (0.6 to 1.1)	1.2 (0.8 to 1.7)	0.5 (0.2 to 0.8)	0.9 (0.6 to 1.3)	1.3 (0.7 to 1.9)	0.5 (0.2 to 0.9)
	2006	0.6 (0.4 to 0.8)	0.9 (0.6 to 1.4)	0.2 (0.0 to 0.4)	0.6 (0.4 to 1.0)	1.1 (0.6 to 1.6)	0.2 (0.0 to 0.5)
	2007	0.7 (0.5 to 0.9)	1.2 (0.8 to 1.7)	0.1 (0.0 to 0.3)	0.7 (0.5 to 1.1)	1.3 (0.8 to 1.9)	0.2 (0.0 to 0.4)
	2008	0.8 (0.5 to 1.1)	1.2 (0.8 to 1.7)	0.3 (0.2 to 0.6)	0.8 (0.5 to 1.3)	1.3 (0.7 to 2.1)	0.4 (0.2 to 0.7)
	2009	0.8 (0.5 to 1.0)	1.3 (0.9 to 1.8)	0.2 (0.1 to 0.4)	0.8 (0.5 to 1.2)	1.4 (0.8 to 2.1)	0.2 (0.1 to 0.6)
	2010	0.9 (0.6 to 1.2)	1.3 (0.9 to 1.8)	0.5 (0.2 to 0.9)	1.0 (0.7 to 1.4)	1.5 (0.9 to 2.2)	0.5 (0.2 to 0.9)
	2011	0.8 (0.6 to 1.1)	1.1 (0.8 to 1.6)	0.4 (0.2 to 0.7)	0.9 (0.6 to 1.3)	1.3 (0.8 to 2.1)	0.4 (0.2 to 0.8)
	2012	1.1 (0.8 to 1.4)	1.7 (1.2 to 2.3)	0.4 (0.2 to 0.7)	1.2 (0.8 to 1.6)	1.8 (1.2 to 2.5)	0.5 (0.2 to 0.9)
	2013	1.1 (0.9 to 1.5)	1.5 (1.1 to 2.1)	0.7 (0.4 to 1.1)	1.2 (0.9 to 1.7)	1.7 (1.1 to 2.5)	0.8 (0.4 to 1.2)
	2014	1.6 (1.2 to 2.0)	2.5 (1.9 to 3.2)	0.6 (0.3 to 1.0)	1.7 (1.2 to 2.2)	2.7 (1.9 to 3.6)	0.6 (0.3 to 1.0)
	2015	1.3 (1.0 to 1.7)	2.0 (1.5 to 2.7)	0.5 (0.3 to 0.8)	1.4 (1.0 to 1.8)	2.2 (1.5 to 2.9)	0.5 (0.3 to 0.9)
Other specified and unspecified types	1993	7.5 (6.7 to 8.3)	11.6 (10.2 to 13.1)	3.8 (3.0 to 4.6)	7.5 (6.7 to 8.3)	11.6 (10.2 to 13.1)	3.8 (3.0 to 4.6)
	1994	6.8 (6.1 to 7.5)	10.3 (9.0 to 11.7)	3.6 (2.9 to 4.4)	6.8 (6.1 to 7.5)	10.3 (9.0 to 11.7)	3.6 (2.9 to 4.4)
	1995	6.7 (6.0 to 7.5)	10.1 (8.9 to 11.4)	3.7 (3.0 to 4.5)	6.7 (6.0 to 7.5)	10.1 (8.9 to 11.4)	3.7 (3.0 to 4.5)
	1996	7.1 (6.4 to 7.9)	10.6 (9.3 to 11.9)	4.0 (3.3 to 4.8)	7.1 (6.4 to 7.9)	10.6 (9.3 to 11.9)	4.0 (3.3 to 4.8)
	1997	6.2 (5.5 to 6.8)	9.4 (8.2 to 10.6)	3.3 (2.7 to 4.0)	6.2 (5.5 to 6.8)	9.4 (8.2 to 10.6)	3.3 (2.7 to 4.0)
	1998	5.5 (4.9 to 6.1)	8.3 (7.2 to 9.4)	2.9 (2.3 to 3.6)	5.5 (4.9 to 6.1)	8.3 (7.2 to 9.4)	2.9 (2.3 to 3.6)
	1999	6.2 (5.6 to 6.9)	8.8 (7.7 to 10.0)	3.9 (3.2 to 4.6)	6.2 (5.6 to 6.9)	8.8 (7.7 to 10.0)	3.9 (3.2 to 4.6)
	2000	6.5 (5.9 to 7.2)	9.3 (8.2 to 10.6)	4.0 (3.3 to 4.7)	6.5 (5.9 to 7.2)	9.3 (8.2 to 10.6)	4.0 (3.3 to 4.7)
	2001	6.1 (5.5 to 6.8)	8.9 (7.9 to 10.1)	3.6 (3.0 to 4.3)	6.1 (5.5 to 6.8)	8.9 (7.9 to 10.1)	3.6 (3.0 to 4.3)
	2002	6.9 (6.2 to 7.6)	10.5 (9.4 to 11.7)	3.5 (2.9 to 4.2)	6.9 (6.2 to 7.6)	10.5 (9.4 to 11.7)	3.5 (2.9 to 4.2)
	2003	6.3 (5.7 to 6.9)	9.8 (8.7 to 11.0)	3.0 (2.5 to 3.7)	6.3 (5.7 to 6.9)	9.8 (8.7 to 11.0)	3.0 (2.5 to 3.7)
	2004	6.7 (6.1 to 7.4)	10.5 (9.4 to 11.7)	3.1 (2.6 to 3.8)	6.7 (6.1 to 7.4)	10.5 (9.4 to 11.7)	3.1 (2.6 to 3.8)
	2005	6.1 (5.5 to 6.7)	9.5 (8.5 to 10.6)	3.0 (2.5 to 3.6)	6.1 (5.5 to 6.7)	9.5 (8.5 to 10.6)	3.0 (2.5 to 3.6)
	2006	6.3 (5.7 to 6.9)	9.3 (8.3 to 10.4)	3.5 (2.9 to 4.1)	6.3 (5.7 to 6.9)	9.3 (8.3 to 10.4)	3.5 (2.9 to 4.1)
	2007	5.4 (4.8 to 5.9)	8.4 (7.5 to 9.5)	2.4 (2.0 to 2.9)	5.4 (4.8 to 5.9)	8.4 (7.5 to 9.5)	2.4 (2.0 to 2.9)
	2008	6.1 (5.5 to 6.7)	9.4 (8.4 to 10.5)	2.9 (2.4 to 3.4)	6.1 (5.5 to 6.7)	9.4 (8.4 to 10.5)	2.9 (2.4 to 3.4)
	2009	5.0 (4.6 to 5.6)	7.4 (6.6 to 8.3)	2.8 (2.4 to 3.4)	5.0 (4.6 to 5.6)	7.4 (6.6 to 8.3)	2.8 (2.4 to 3.4)
	2010	5.2 (4.7 to 5.8)	7.6 (6.7 to 8.5)	3.0 (2.5 to 3.6)	5.2 (4.7 to 5.8)	7.6 (6.7 to 8.5)	3.0 (2.5 to 3.6)
	2011	5.1 (4.6 to 5.6)	7.4 (6.5 to 8.3)	2.8 (2.3 to 3.4)	5.1 (4.6 to 5.6)	7.4 (6.5 to 8.3)	2.8 (2.3 to 3.4)
	2012	4.8 (4.3 to 5.3)	7.0 (6.2 to 8.0)	2.5 (2.0 to 3.0)	4.8 (4.3 to 5.3)	7.0 (6.2 to 8.0)	2.5 (2.0 to 3.0)
	2013	4.8 (4.3 to 5.4)	7.2 (6.3 to 8.1)	2.5 (2.0 to 3.0)	4.8 (4.3 to 5.4)	7.2 (6.3 to 8.1)	2.5 (2.0 to 3.0)
	2014	5.0 (4.5 to 5.6)	7.2 (6.4 to 8.0)	2.9 (2.4 to 3.5)	5.0 (4.5 to 5.6)	7.2 (6.4 to 8.0)	2.9 (2.4 to 3.5)

Histological type	Year at diagnosis	Completed ASIRs (per 100,000 person-years)			Imputed ASIRs (per 100,000 person-years)		
		**Overall**	**Male**	**Female**	**Overall**	**Male**	**Female**
	2015	4.4 (4.0 to 4.9)	6.2 (5.4 to 7.0)	2.6 (2.1 to 3.1)	4.4 (4.0 to 4.9)	6.2 (5.4 to 7.0)	2.6 (2.1 to 3.1)

Notes: ASIRs=Age-standardized incidence rates expressed per 100,000 person-years using the "Standard Japanese Population in 1985"; 95% CIs=95% Confidence intervals;
Three Japanese cancer registries are in Yamagata, Fukui and Nagasaki prefectures.

Table S6: Completed and imputed ASIRs of lung cancer in three cancer registries in Japan, 1993–2015, by stage at diagnosis

Stage at diagnosis	Year at diagnosis	Completed ASIRs (per 100,000 person-years)			Imputed ASIRs (per 100,000 person-years)		
		Overall	Male	Female	Overall	Male	Female
Localized	1993	7.3 (6.5 to 8.2)	10.3 (9.0 to 11.7)	4.6 (3.7 to 5.6)	8.8 (7.4 to 10.2)	12.4 (10.1 to 14.8)	5.4 (4.3 to 6.8)
	1994	7.3 (6.5 to 8.1)	10.3 (9.0 to 11.7)	4.5 (3.7 to 5.5)	8.6 (7.5 to 9.8)	12.1 (10.2 to 14.2)	5.3 (4.1 to 6.7)
	1995	6.8 (6.0 to 7.6)	8.9 (7.7 to 10.2)	4.7 (3.9 to 5.7)	8.3 (6.9 to 9.8)	11.1 (8.6 to 13.9)	5.7 (4.4 to 7.2)
	1996	8.0 (7.2 to 8.8)	10.6 (9.3 to 12.1)	5.5 (4.6 to 6.5)	9.3 (8.2 to 10.6)	12.6 (10.8 to 14.7)	6.2 (4.9 to 7.7)
	1997	8.8 (7.9 to 9.7)	11.8 (10.4 to 13.3)	5.8 (4.9 to 6.9)	10.4 (9.2 to 11.6)	14.2 (12.1 to 16.5)	6.7 (5.4 to 8.2)
	1998	8.6 (7.8 to 9.5)	11.2 (9.9 to 12.6)	6.1 (5.1 to 7.2)	10.0 (8.9 to 11.3)	13.3 (11.4 to 15.3)	7.0 (5.7 to 8.5)
	1999	9.5 (8.7 to 10.5)	11.9 (10.5 to 13.3)	7.4 (6.3 to 8.6)	11.2 (9.9 to 12.5)	14.0 (12.0 to 16.2)	8.6 (7.2 to 10.1)
	2000	9.5 (8.6 to 10.4)	11.7 (10.4 to 13.1)	7.4 (6.3 to 8.6)	11.1 (9.9 to 12.3)	14.0 (12.2 to 15.9)	8.3 (6.9 to 9.8)
	2001	8.7 (7.9 to 9.6)	11.1 (9.8 to 12.4)	6.5 (5.5 to 7.5)	10.5 (9.2 to 11.8)	13.6 (11.3 to 16.1)	7.4 (6.1 to 9.0)
	2002	9.4 (8.6 to 10.3)	11.8 (10.5 to 13.2)	7.1 (6.0 to 8.2)	11.4 (10.2 to 12.8)	14.7 (12.8 to 16.8)	8.3 (6.8 to 9.9)
	2003	9.9 (9.1 to 10.8)	11.6 (10.3 to 12.9)	8.3 (7.2 to 9.5)	11.9 (10.7 to 13.2)	14.2 (12.4 to 16.3)	9.6 (8.1 to 11.3)
	2004	10.7 (9.8 to 11.6)	12.6 (11.3 to 14.1)	8.7 (7.5 to 9.9)	12.3 (11.0 to 13.6)	15.0 (12.9 to 17.2)	9.6 (8.3 to 11.0)
	2005	10.2 (9.4 to 11.1)	12.3 (11.0 to 13.6)	8.2 (7.1 to 9.4)	11.6 (10.3 to 13.0)	14.1 (12.1 to 16.4)	9.1 (7.8 to 10.4)
	2006	11.9 (10.9 to 12.8)	13.7 (12.4 to 15.2)	9.9 (8.7 to 11.2)	13.1 (11.9 to 14.3)	15.5 (13.7 to 17.4)	10.6 (9.2 to 12.2)
	2007	12.4 (11.4 to 13.4)	15.7 (14.2 to 17.2)	8.9 (7.8 to 10.2)	13.4 (12.2 to 14.7)	17.2 (15.3 to 19.2)	9.5 (8.2 to 11.0)
	2008	13.7 (12.7 to 14.7)	15.7 (14.3 to 17.3)	11.4 (10.1 to 12.9)	14.7 (13.6 to 16.0)	17.3 (15.5 to 19.2)	12.1 (10.6 to 13.7)
	2009	14.5 (13.5 to 15.6)	16.3 (14.8 to 17.9)	12.5 (11.2 to 13.9)	15.2 (14.0 to 16.5)	17.3 (15.4 to 19.4)	13.0 (11.4 to 14.6)
	2010	15.9 (14.8 to 17.0)	18.0 (16.5 to 19.7)	13.6 (12.2 to 15.1)	16.6 (15.4 to 17.9)	19.0 (17.2 to 21.0)	14.0 (12.4 to 15.8)
	2011	15.6 (14.5 to 16.6)	18.5 (16.9 to 20.1)	12.4 (11.0 to 13.8)	16.3 (15.0 to 17.7)	19.4 (17.6 to 21.4)	13.0 (11.4 to 14.7)
	2012	16.9 (15.8 to 18.1)	18.7 (17.2 to 20.4)	14.7 (13.2 to 16.3)	17.5 (16.3 to 18.8)	19.5 (17.8 to 21.4)	15.1 (13.5 to 16.8)
	2013	17.7 (16.6 to 18.8)	20.0 (18.4 to 21.7)	15.1 (13.6 to 16.7)	18.3 (17.1 to 19.6)	20.9 (19.1 to 22.8)	15.4 (13.8 to 17.0)
	2014	18.3 (17.2 to 19.5)	20.3 (18.7 to 22.1)	15.9 (14.3 to 17.5)	18.8 (17.6 to 20.1)	21.1 (19.3 to 23.0)	16.1 (14.6 to 17.8)
	2015	16.5 (15.4 to 17.6)	18.4 (16.9 to 20.1)	14.2 (12.7 to 15.8)	17.1 (15.9 to 18.3)	19.2 (17.5 to 21.1)	14.5 (13.0 to 16.2)
Regional	1993	10.6 (9.7 to 11.6)	17.1 (15.4 to 18.9)	4.6 (3.8 to 5.6)	14.7 (12.5 to 17.0)	23.5 (19.9 to 27.4)	6.5 (4.5 to 8.8)
	1994	10.6 (9.7 to 11.6)	17.1 (15.4 to 18.9)	4.6 (3.8 to 5.6)	14.3 (12.8 to 15.8)	23.1 (20.2 to 26.2)	6.1 (4.6 to 7.9)
	1995	11.0 (10.0 to 12.0)	17.5 (15.8 to 19.4)	4.9 (4.0 to 5.9)	14.9 (12.9 to 17.1)	23.9 (20.1 to 28.0)	6.7 (5.3 to 8.3)
	1996	11.4 (10.4 to 12.4)	18.1 (16.3 to 19.9)	5.0 (4.2 to 6.0)	15.1 (13.4 to 17.0)	24.0 (20.8 to 27.5)	6.8 (5.5 to 8.3)
	1997	11.3 (10.4 to 12.3)	17.6 (15.9 to 19.4)	5.3 (4.4 to 6.3)	15.4 (13.9 to 17.1)	24.1 (21.1 to 27.3)	7.2 (5.8 to 8.9)
	1998	11.2 (10.3 to 12.2)	17.5 (15.8 to 19.2)	5.2 (4.3 to 6.2)	15.2 (13.5 to 17.1)	24.0 (20.7 to 27.6)	7.0 (5.6 to 8.5)
	1999	11.4 (10.4 to 12.4)	18.1 (16.5 to 19.9)	4.9 (4.1 to 5.9)	15.4 (13.6 to 17.4)	24.7 (21.6 to 28.2)	6.6 (5.1 to 8.4)
	2000	9.7 (8.8 to 10.6)	15.8 (14.2 to 17.4)	3.7 (3.0 to 4.5)	13.3 (11.6 to 15.2)	21.7 (18.3 to 25.3)	5.3 (3.8 to 7.1)
	2001	10.5 (9.6 to 11.4)	15.9 (14.4 to 17.6)	5.1 (4.2 to 6.1)	14.4 (12.5 to 16.4)	22.3 (19.1 to 25.7)	6.8 (5.1 to 8.8)
	2002	11.3 (10.4 to 12.3)	16.9 (15.3 to 18.5)	5.9 (4.9 to 6.9)	15.6 (13.8 to 17.5)	23.9 (20.9 to 27.2)	7.5 (5.8 to 9.5)
	2003	11.7 (10.8 to 12.6)	18.1 (16.5 to 19.8)	5.4 (4.5 to 6.3)	15.5 (14.0 to 17.2)	24.2 (21.3 to 27.3)	7.1 (5.7 to 8.7)
	2004	11.6 (10.7 to 12.6)	16.9 (15.4 to 18.5)	6.4 (5.5 to 7.4)	15.2 (13.7 to 16.8)	22.7 (20.2 to 25.3)	7.9 (6.5 to 9.4)
	2005	12.2 (11.3 to 13.1)	18.6 (17.0 to 20.3)	5.8 (4.9 to 6.7)	15.1 (13.8 to 16.5)	23.1 (20.6 to 25.8)	7.2 (5.8 to 8.7)
	2006	11.9 (11.0 to 12.9)	18.0 (16.4 to 19.6)	5.8 (4.9 to 6.8)	14.6 (13.2 to 16.0)	22.0 (19.8 to 24.5)	7.1 (5.7 to 8.7)
	2007	12.0 (11.1 to 13.0)	19.0 (17.3 to 20.7)	4.8 (4.0 to 5.7)	14.1 (12.8 to 15.4)	22.2 (20.1 to 24.5)	5.7 (4.5 to 7.1)
	2008	12.3 (11.4 to 13.3)	18.6 (17.1 to 20.3)	5.7 (4.9 to 6.7)	14.4 (13.2 to 15.7)	21.9 (19.8 to 24.2)	6.6 (5.3 to 8.1)
	2009	12.5 (11.6 to 13.5)	19.0 (17.5 to 20.7)	5.6 (4.7 to 6.5)	14.0 (12.9 to 15.3)	21.4 (19.3 to 23.7)	6.3 (5.2 to 7.6)
	2010	12.7 (11.8 to 13.7)	19.4 (17.8 to 21.1)	5.7 (4.8 to 6.7)	13.9 (12.7 to 15.1)	21.0 (19.0 to 23.2)	6.4 (5.2 to 7.7)
	2011	12.0 (11.1 to 13.0)	17.4 (15.9 to 19.0)	6.2 (5.3 to 7.3)	13.3 (12.0 to 14.8)	19.4 (16.9 to 22.0)	6.9 (5.7 to 8.1)
	2012	11.0 (10.1 to 12.0)	16.9 (15.4 to 18.5)	4.8 (4.0 to 5.7)	12.1 (11.0 to 13.4)	18.6 (16.6 to 20.7)	5.3 (4.2 to 6.5)
	2013	10.3 (9.4 to 11.2)	14.9 (13.5 to 16.4)	5.2 (4.4 to 6.2)	11.2 (10.0 to 12.4)	16.3 (14.3 to 18.5)	5.6 (4.7 to 6.7)
	2014	10.2 (9.3 to 11.1)	15.4 (14.0 to 17.0)	4.4 (3.6 to 5.2)	11.0 (9.8 to 12.2)	16.6 (14.7 to 18.6)	4.7 (3.8 to 5.9)

Stage at diagnosis	Year at diagnosis	Completed ASIRs (per 100,000 person-years)			Imputed ASIRs (per 100,000 person-years)		
		Overall	Male	Female	Overall	Male	Female
	2015	9.4 (8.6 to 10.3)	14.6 (13.2 to 16.1)	3.7 (3.0 to 4.5)	10.2 (9.2 to 11.3)	15.8 (14.1 to 17.6)	4.1 (3.2 to 5.0)
Distant	1993	11.2 (10.3 to 12.3)	17.4 (15.6 to 19.2)	5.5 (4.6 to 6.5)	17.9 (15.6 to 20.3)	26.9 (23.0 to 31.2)	9.5 (7.8 to 11.5)
	1994	11.1 (10.2 to 12.2)	16.3 (14.6 to 18.0)	6.4 (5.4 to 7.5)	17.7 (16.2 to 19.3)	25.8 (22.9 to 28.9)	10.2 (8.4 to 12.2)
	1995	11.9 (10.9 to 12.9)	17.9 (16.2 to 19.7)	6.3 (5.3 to 7.4)	18.8 (17.0 to 20.7)	28.0 (24.8 to 31.5)	10.2 (8.6 to 12.0)
	1996	11.8 (10.8 to 12.8)	17.1 (15.5 to 18.9)	6.7 (5.7 to 7.9)	18.4 (16.6 to 20.3)	26.6 (23.5 to 29.8)	10.8 (9.2 to 12.6)
	1997	11.9 (10.9 to 12.9)	18.1 (16.4 to 19.9)	5.9 (4.9 to 6.9)	19.5 (17.7 to 21.5)	29.8 (26.3 to 33.4)	9.9 (8.4 to 11.5)
	1998	10.8 (9.8 to 11.7)	16.7 (15.1 to 18.5)	5.0 (4.2 to 6.0)	17.7 (15.9 to 19.6)	26.8 (23.5 to 30.3)	9.2 (7.5 to 11.0)
	1999	11.4 (10.5 to 12.4)	17.8 (16.1 to 19.5)	5.4 (4.5 to 6.4)	18.3 (16.4 to 20.3)	28.1 (24.7 to 31.8)	9.1 (7.5 to 10.9)
	2000	11.3 (10.4 to 12.3)	16.6 (15.0 to 18.3)	6.2 (5.2 to 7.2)	18.6 (16.7 to 20.6)	27.5 (24.1 to 31.2)	10.1 (8.0 to 12.3)
	2001	12.1 (11.1 to 13.1)	17.7 (16.1 to 19.4)	6.6 (5.6 to 7.6)	19.2 (17.4 to 21.2)	28.3 (25.1 to 31.7)	10.5 (8.8 to 12.4)
	2002	10.5 (9.6 to 11.4)	14.6 (13.2 to 16.2)	6.4 (5.4 to 7.4)	17.8 (16.0 to 19.6)	25.9 (22.9 to 29.0)	9.9 (8.4 to 11.6)
	2003	12.2 (11.2 to 13.1)	17.9 (16.3 to 19.6)	6.4 (5.5 to 7.4)	19.2 (17.5 to 20.9)	28.6 (25.8 to 31.6)	10.0 (8.2 to 12.0)
	2004	13.3 (12.3 to 14.3)	19.7 (18.1 to 21.5)	6.9 (5.9 to 8.0)	19.9 (18.2 to 21.7)	29.6 (26.9 to 32.6)	10.4 (8.8 to 12.2)
	2005	12.4 (11.5 to 13.4)	20.1 (18.4 to 21.8)	4.8 (4.0 to 5.7)	18.2 (16.6 to 19.8)	27.9 (25.2 to 30.8)	8.6 (7.1 to 10.3)
	2006	13.6 (12.6 to 14.6)	20.0 (18.4 to 21.8)	7.0 (6.0 to 8.0)	19.3 (17.8 to 20.9)	28.1 (25.6 to 30.8)	10.4 (8.8 to 12.2)
	2007	14.0 (13.0 to 15.0)	20.7 (19.0 to 22.5)	7.1 (6.1 to 8.1)	18.4 (16.9 to 20.0)	27.3 (24.9 to 29.9)	9.3 (8.0 to 10.9)
	2008	15.5 (14.4 to 16.6)	23.2 (21.4 to 25.1)	7.5 (6.5 to 8.6)	20.1 (18.7 to 21.7)	30.0 (27.6 to 32.5)	10.0 (8.6 to 11.6)
	2009	16.7 (15.7 to 17.9)	23.9 (22.1 to 25.8)	9.3 (8.1 to 10.5)	20.3 (18.8 to 21.9)	28.8 (26.2 to 31.5)	11.5 (10.0 to 13.1)
	2010	17.1 (16.0 to 18.2)	24.9 (23.1 to 26.9)	8.8 (7.7 to 10.0)	20.4 (19.0 to 21.8)	29.5 (27.2 to 31.8)	10.8 (9.5 to 12.2)
	2011	17.0 (15.9 to 18.1)	23.9 (22.1 to 25.8)	9.5 (8.4 to 10.8)	20.5 (18.9 to 22.1)	28.7 (25.8 to 31.7)	11.7 (10.2 to 13.3)
	2012	18.1 (16.9 to 19.3)	25.1 (23.2 to 27.1)	10.5 (9.3 to 11.8)	21.2 (19.8 to 22.7)	29.3 (27.1 to 31.7)	12.5 (11.0 to 14.2)
	2013	17.1 (16.0 to 18.2)	24.9 (23.1 to 26.8)	8.7 (7.7 to 9.9)	19.7 (18.3 to 21.1)	28.5 (26.2 to 30.9)	10.2 (8.9 to 11.5)
	2014	17.3 (16.2 to 18.5)	24.6 (22.8 to 26.5)	9.4 (8.3 to 10.6)	19.8 (18.5 to 21.2)	28.2 (25.9 to 30.5)	10.8 (9.4 to 12.3)
	2015	16.9 (15.9 to 18.1)	23.4 (21.6 to 25.2)	9.8 (8.6 to 11.1)	19.3 (18.0 to 20.7)	26.8 (24.7 to 29.2)	11.0 (9.7 to 12.5)

Notes: ASIRs=Age-standardized incidence rates expressed per 100,000 person-years using the "Standard Japanese Population in 1985"; 95% CIs=95% Confidence intervals
Three Japanese cancer registries are in Yamagata, Fukui and Nagasaki prefectures.

Table S7: Joinpoint regression analysis of trends in completed ASIRs (before imputation) of lung cancer in three cancer registries in Japan, 1993–2015, by histological type and stage at diagnosis separately.

	Categories	AAPC (95% CI)	Trends	Trend 1		Trend 2		Trend 3	
				Period_1	APC (95% CI)_1	Period_2	APC (95% CI)_2	Period_3	APC (95% CI)_3
Male	Total	0.0 (-0.4 to 0.4)	2	1993–2011	0.5 (0.3 to 0.7)***	2011–2015	-2.1 (-4.2 to 0.0)		
	Small cell carcinoma	-0.3 (-0.8 to 0.2)	1	1993–2015	-0.3 (-0.8 to 0.2)				
	Squamous cell carcinoma	-0.8 (-1.2 to -0.4)***	1	1993–2015	-0.8 (-1.2 to -0.4)***				
	Adenocarcinoma	1.9 (1.6 to 2.3)***	1	1993–2015	1.9 (1.6 to 2.3)***				
	Localized	3.0 (1.8 to 4.2)***	3	1993–2004	1.9 (0.6 to 3.1)**	2004–2012	6.1 (4.1 to 8.1)***	2012–2015	-0.8 (-6.9 to 5.6)
	Regional	-0.7 (-1.5 to 0.0)	2	1993–2010	0.6 (0.1 to 1.1)*	2010–2015	-5.1 (-8.0 to -2.2)**		
	Distant	1.5 (0.7 to 2.2)***	3	1993–2002	-0.4 (-1.5 to 0.7)	2002–2010	5.1 (3.6 to 6.5)***	2010–2015	-0.8 (-2.9 to 1.3)
Female	Total	1.9 (1.5 to 2.2)***	1	1993–2015	1.9 (1.5 to 2.2)***				
	Small cell carcinoma	0.8 (-0.2 to 1.9)	1	1993–2015	0.8 (-0.2 to 1.9)				
	Squamous cell carcinoma	0.5 (-0.6 to 1.7)	1	1993–2015	0.5 (-0.6 to 1.7)				
	Adenocarcinoma	3.4 (2.9 to 3.8)***	1	1993–2015	3.4 (2.9 to 3.8)***				
	Localized	6.0 (5.4 to 6.6)***	1	1993–2015	6.0 (5.4 to 6.6)***				
	Regional	-0.9 (-2.8 to 1.0)	2	1993–2011	1.3 (0.3 to 2.3)*	2011–2015	-10.3 (-19.2 to -0.5)*		
	Distant	2.8 (1.5 to 4.0)***	2	1993–2005	0.9 (-0.8 to 2.7)	2005–2015	5.0 (3.0 to 7.1)***		

Notes: ASIRs=Age-standardized incidence rates; AAPC=Average annual percentage change; APC=Annual percentage change; CI=Confidence Interval; ***, **, * Statistically significant results with P<0.001, P<0.01, and P<0.05, respectively; Three Japanese cancer registries are in Yamagata, Fukui and Nagasaki prefectures.

Table S8: Joinpoint regression analysis of trends in completed ASIRs (before imputation) of lung cancer in three cancer registries in Japan, 1993–2015, by histology-stage-combination

	Histological type	Stage at diagnosis	AAPC (95% CI)	Trends	Trend 1		Trend 2		Trend 3	
					Period	APC (95% CI)	Period	APC (95% CI)	Period	APC (95% CI)
Male	Small cell carcinoma	Localized	0.1 (-1.7 to 1.9)	1	1993–2015	0.1 (-1.7 to 1.9)				
		Regional	-0.6 (-1.6 to 0.3)	1	1993–2015	-0.6 (-1.6 to 0.3)				
		Distant	1.8 (0.9 to 2.6)***	1	1993–2015	1.8 (0.9 to 2.6)***				
	Squamous cell carcinoma	Localized	0.2 (-1.5 to 1.9)	2	1993–2003	-2.7 (-5.6 to 0.4)	2003–2015	2.7 (0.6 to 4.8)*		
		Regional	-1.0 (-1.5 to -0.4)**	1	1993–2015	-1.0 (-1.5 to -0.4)**				
		Distant	0.3 (-1.8 to 2.4)	3	1993–2005	-1.2 (-2.8 to 0.4)	2005–2010	10.3 (2.2 to 18.9)*	2010–2015	-5.5 (-10.3 to -0.5)*
	Adenocarcinoma	Localized	5.1 (4.6 to 5.7)***	1	1993–2015	5.1 (4.6 to 5.7)***				
		Regional	-0.9 (-2.0 to 0.2)	2	1993–2009	1.3 (0.4 to 2.2)**	2009–2015	-6.7 (-10.1 to -3.1)**		
		Distant	2.8 (2.2 to 3.4)***	1	1993–2015	2.8 (2.2 to 3.4)***				
Female	Small cell carcinoma	Localized	-2.4 (-5.1 to 0.5)	1	1993–2015	-2.4 (-5.1 to 0.5)				
		Regional	-0.5 (-2.7 to 1.8)	1	1993–2015	-0.5 (-2.7 to 1.8)				
		Distant	3.6 (0.6 to 6.6)*	2	1993–1999	-5.4 (-14.6 to 4.8)	1999–2015	7.1 (5.1 to 9.3)***		
	Squamous cell carcinoma	Localized	2.1 (0.3 to 3.9)*	1	1993–2015	2.1 (0.3 to 3.9)*				
		Regional	1.8 (0.1 to 3.6)*	1	1993–2015	1.8 (0.1 to 3.6)*				
		Distant	1.6 (-0.4 to 3.6)	1	1993–2015	1.6 (-0.4 to 3.6)				
	Adenocarcinoma	Localized	6.3 (5.6 to 7.1)***	1	1993–2015	6.3 (5.6 to 7.1)***				
		Regional	-1.6 (-4.2 to 1.1)	2	1993–2011	1.3 (-0.0 to 2.6)	2011–2015	-13.5 (-25.4 to 0.2)		
		Distant	3.0 (1.2 to 4.9)**	2	1993–2003	0.1 (-3.2 to 3.5)	2003–2015	5.5 (3.3 to 7.8)***		

Notes: ASIRs=Age-standardized incidence rates; AAPC=Average annual percentage change; APC=Annual percentage change; CI=Confidence Interval; ***, **, * Statistically significant results with P<0.001, P<0.01, and P<0.05, respectively; Three Japanese cancer registries are in Yamagata, Fukui and Nagasaki prefectures.

Table S9: Kaplan-Meier log-rank test for equality in survival probability between the completed and imputed data in three cancer registries in Japan, 1993–2015, by stage at diagnosis

Year at diagnosis	Stage at diagnosis (P-values from Kaplan-Meier log-rank test)		
	Localized	Regional	Distant
1993	1.000	0.940	0.772
1994	1.000	0.965	0.526
1995	1.000	0.890	0.547
1996	1.000	0.576	0.808
1997	1.000	0.821	0.683
1998	0.995	0.605	0.897
1999	0.997	0.802	0.855
2000	0.990	0.643	0.496
2001	0.910	0.841	0.615
2002	0.962	0.667	0.724
2003	0.974	0.824	0.762
2004	0.867	0.681	0.616
2005	0.753	0.970	0.908
2006	0.912	0.927	0.907
2007	0.993	0.953	0.810
2008	0.944	0.870	0.665
2009	0.970	0.971	0.847
2010	0.985	0.993	0.690
2011	0.988	0.973	0.945
2012	0.987	0.962	0.971
2013	0.987	0.978	0.975
2014	0.858	0.916	0.658
2015	0.519	0.842	0.245

Notes: Three Japanese cancer registries are in Yamagata, Fukui and Nagasaki prefectures.

Table S10: Kaplan-Meier log-rank test for equality in survival probability between the completed and imputed data in three cancer registries in Japan, 1993–2015, by histological type

Year at diagnosis	Histological type (P-values from Kaplan-Meier log-rank test)					
	**Small Cell Carcinoma**	**Squamous cell carcinoma**	**Adenocarcinoma**	**Large cell carcinoma**	**Other non–small cell carcinoma**	**Other specified and unspecified types**
1993	1.000	1.000	0.993	1.000	1.000	1.000
1994	1.000	1.000	1.000	1.000	1.000	1.000
1995	1.000	1.000	0.999	1.000	1.000	1.000
1996	1.000	1.000	0.998	1.000	1.000	1.000
1997	1.000	1.000	0.992	1.000	1.000	1.000
1998	1.000	1.000	1.000	1.000	1.000	1.000
1999	1.000	1.000	0.994	1.000	1.000	1.000
2000	1.000	0.999	0.973	1.000	1.000	1.000
2001	0.999	1.000	0.907	1.000	1.000	1.000
2002	0.999	1.000	0.973	1.000	1.000	1.000
2003	1.000	0.999	0.847	1.000	1.000	1.000
2004	1.000	0.998	0.793	1.000	1.000	1.000
2005	1.000	0.999	0.829	1.000	1.000	1.000
2006	1.000	1.000	0.863	1.000	1.000	1.000
2007	1.000	0.996	0.711	1.000	1.000	1.000
2008	1.000	1.000	0.719	1.000	1.000	1.000
2009	0.999	1.000	0.538	1.000	1.000	1.000
2010	1.000	0.992	0.397	1.000	1.000	1.000
2011	1.000	0.998	0.604	1.000	1.000	1.000
2012	1.000	0.999	0.323	1.000	1.000	1.000
2013	1.000	0.985	0.097	1.000	1.000	1.000
2014	0.999	0.867	0.093	1.000	1.000	1.000
2015	0.988	0.793	0.152	1.000	1.000	1.000

Notes: Three Japanese cancer registries are in Yamagata, Fukui and Nagasaki prefectures.

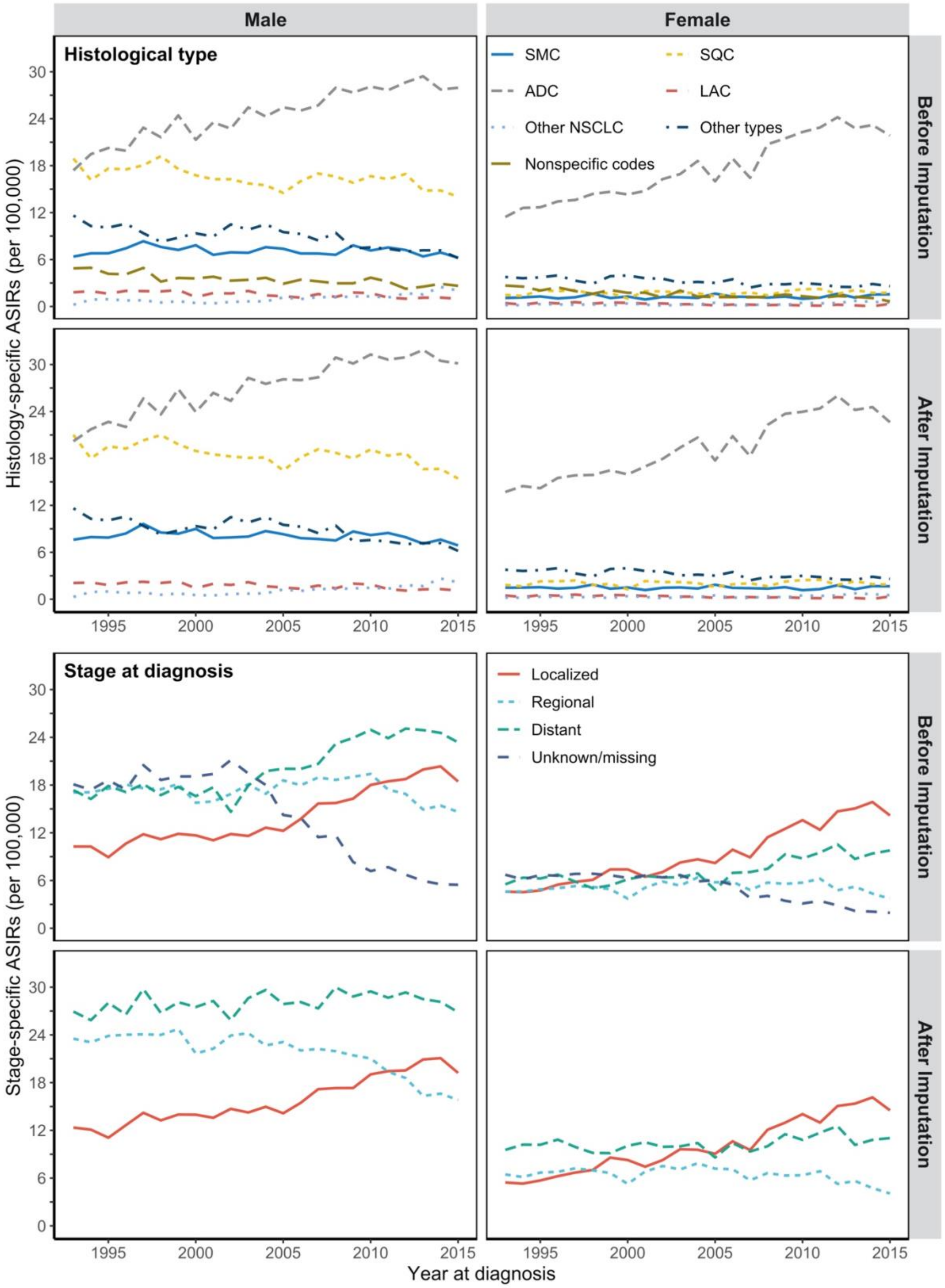

Figure S1: Completed and imputed ASIRs (before and after imputation) of lung cancer in three cancer registries in Japan, 1993–2015, by histological type and stage at diagnosis

Notes: ASIRs=Age-standardized incidence rates; SMC=Small cell carcinoma; SQC=Squamous cell carcinoma; ADC=Adenocarcinoma; The solid lines are the completed trends; The dashed lines are the imputed trends; The shaded areas are the 95% Confidence Intervals; Three Japanese cancer registries are in Yamagata, Fukui and Nagasaki prefectures.

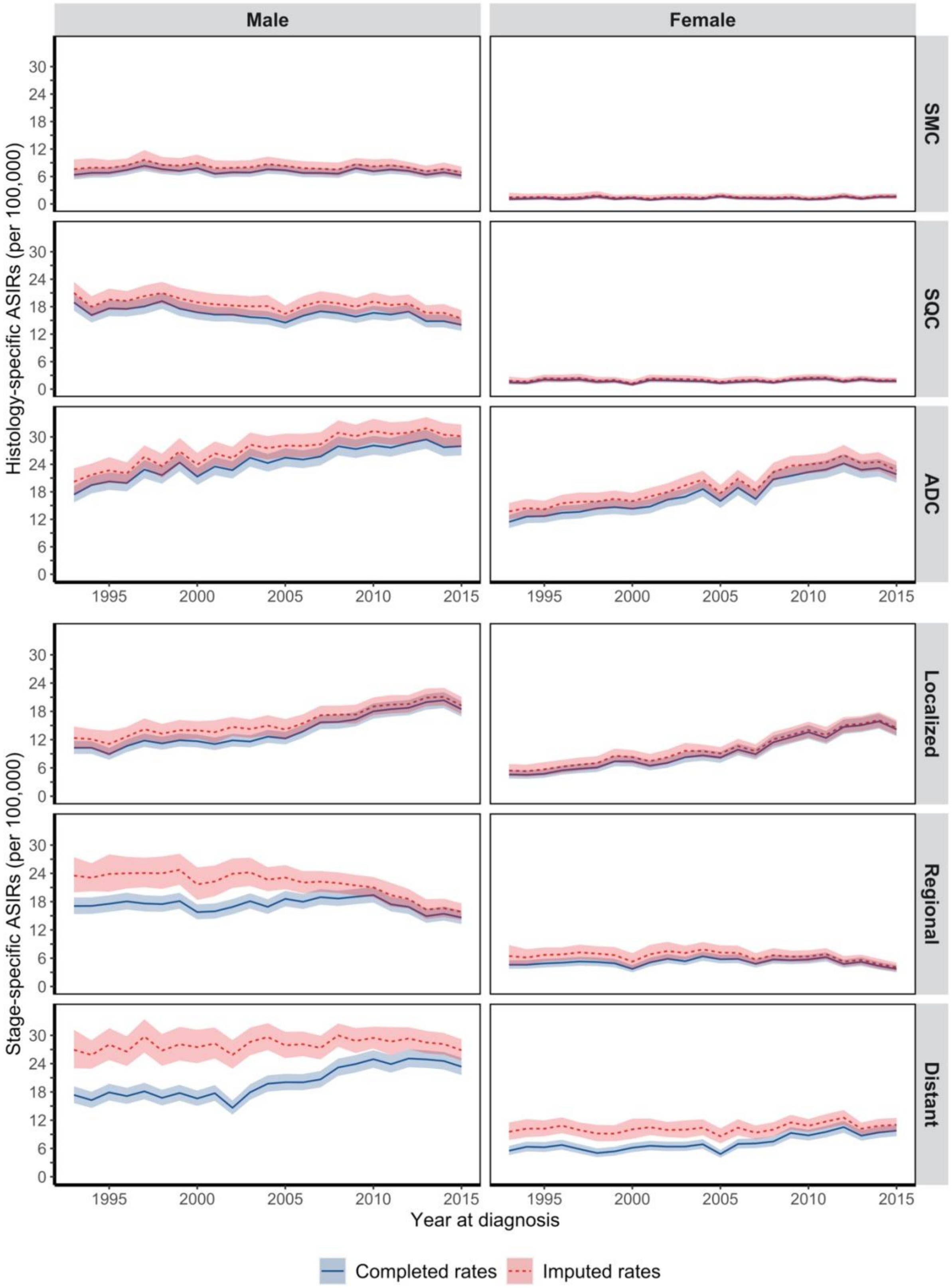

Figure S2: Histology- and stage-specific ASIRs of lung cancer in three cancer registries in Japan, 1993–2015, separately by completed and imputed data (before and after imputation)

Notes: ASIRs=Age-standardized incidence rates; SMC=Small cell carcinoma; SQC=Squamous cell carcinoma; ADC=Adenocarcinoma, LAC=Large cell carcinoma; Other NSCLC=Other non-small cell lung cancer; Other types=Other specified and unspecified types. The three Japanese cancer registries are in Yamagata, Fukui and Nagasaki prefectures.

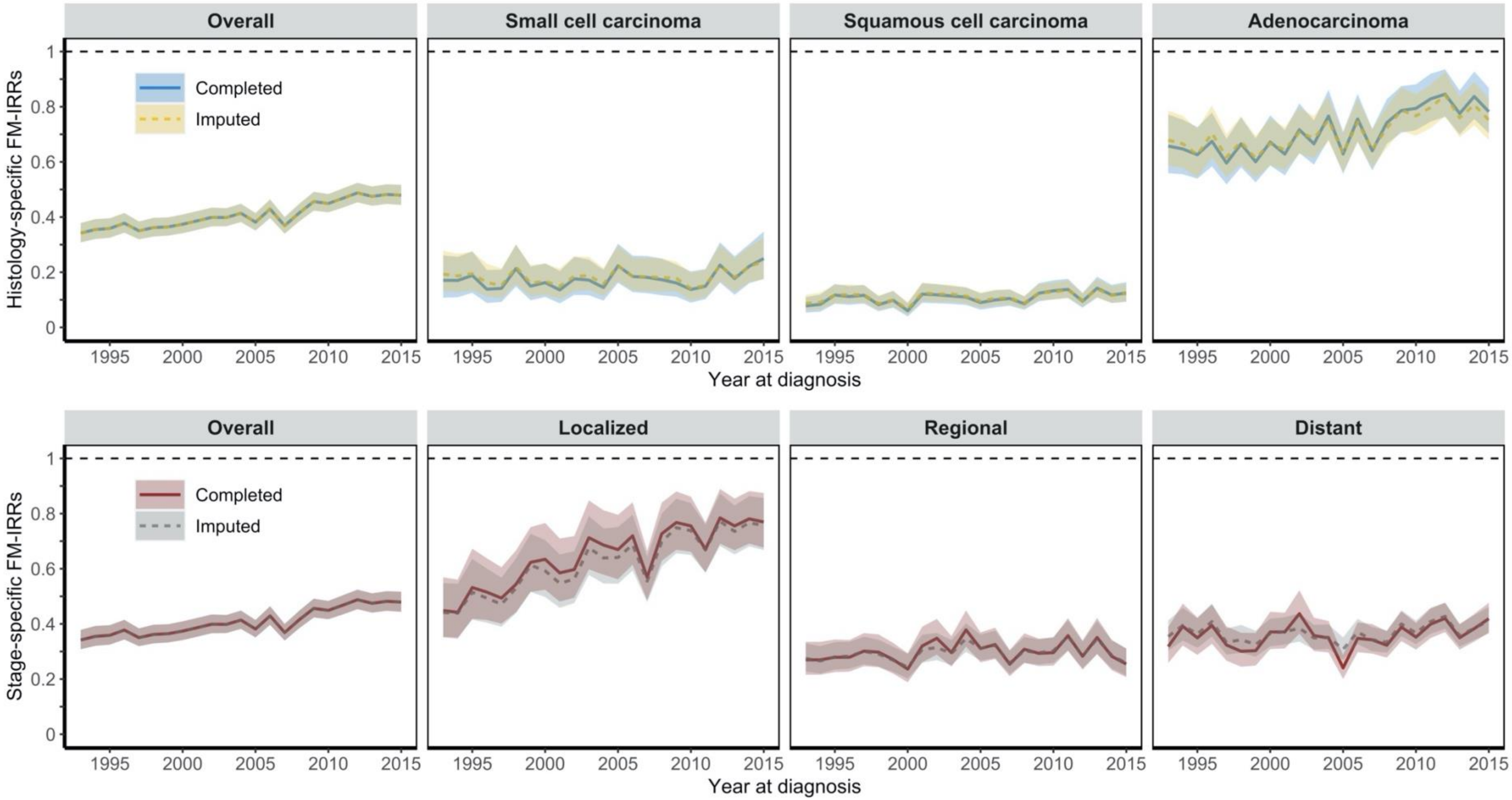

Figure S3: Completed and imputed female-to-male IRRs (before and after imputation) of lung cancer in three cancer registries in Japan, 1993–2015, by histological type and stage at diagnosis separately

Notes: IRRs=Incidence rate ratios; The solid lines are the completed trends; The dashed lines are the imputed trends; The shaded areas are the 95% Confidence Intervals; Three Japanese cancer registries are in Yamagata, Fukui and Nagasaki prefectures.
